# A shift away from mutualism under food-deprived conditions in an anemone-dinoflagellate association

**DOI:** 10.7717/peerj.9745

**Published:** 2020-10-28

**Authors:** Shao-En Peng, Alessandro Moret, Cherilyn Chang, Anderson B. Mayfield, Yu-Ting Ren, Wan-Nan U. Chen, Mario Giordano, Chii-Shiarng Chen

**Affiliations:** 1 National Museum of Marine Biology and Aquarium, Pingtung, Taiwan; 2 Graduate Institute of Marine Biology, National Dong Hwa University, Pingtung, Taiwan; 3 Dipatimento di Scienze della Vita e dell’Ambiente, Università Politecnica delle Marche, Ancona, Italy; 4 Cooperative Institute for Marine and Atmospheric Sciences Studies, University of Miami, Miami, FL, United States of America; 5 Atlantic Oceanographic and Meteorological Laboratory, National Oceanic and Atmospheric Administration, Miami, FL, United States of America; 6 Department of Biological Science and Technology, I-Shou University, Kaohsiung, Taiwan; 7 Institute of Microbiology ASCR, Algatech, Trebon, Czech Republic

**Keywords:** Anemone, *Exaiptasia pallida*, Parasitism, Dinoflagellate, Mutualism, Starvation

## Abstract

The mutualistic symbiosis between anthozoans and intra-gastrodermal dinoflagellates of the family Symbiodiniaceae is the functional basis of all coral reef ecosystems, with the latter providing up to 95% of their fixed photosynthate to their hosts in exchange for nutrients. However, recent studies of sponges, jellyfish, and anemones have revealed the potential for this mutualistic relationship to shift to parasitism under stressful conditions. Over a period of eight weeks, we compared the physiological conditions of both inoculated and aposymbiotic anemones (*Exaiptasia pallida*) that were either fed or starved. By the sixth week, both fed groups of anemones were significantly larger than their starved counterparts. Moreover, inoculated and starved anemones tended to disintegrate into “tissue balls” within eight weeks, and 25% of the samples died; in contrast, starved aposymbiotic anemones required six months to form tissue balls, and no anemones from this group died. Our results show that the dinoflagellates within inoculated anemones may have posed a fatal metabolic burden on their hosts during starvation; this may be because of the need to prioritize their own metabolism and nourishment at the expense of their hosts. Collectively, our study reveals the potential of this dynamic symbiotic association to shift away from mutualism during food-deprived conditions.

## Introduction

The symbiosis between corals and photosynthetic dinoflagellates of the family Symbiodiniaceae allows for these cnidarians to construct reefs across Earth’s tropical seas (Wood, 1999; Stanley, 2003); prior to the emergence of these symbioses, although anthozoans were well diversified, they did not play a major role in reef construction (Gaetani & Gorza, 1989; Stanley & Swart, 1995; Russo et al., 1997; Russo et al., 2000; Keim & Schlager, 2001). Given the recent threats of climate change towards coral reef ecosystems, researchers have been making a concerted effort to better understand the fundamental biology of anthozoan-dinoflagellate symbioses (e.g., Mayfield & Gates, 2007; Mayfield et al., 2014), as well as why they bleach upon prolonged exposure to unfavorable environmental conditions (Mayfield et al., 2013; Mayfield, Fan & Chen, 2013). Unfortunately, investigations of the symbiotic relationship between hermatypic corals and Symbiodiniaceae dinoflagellates have been hampered by the slow growth of most corals, the difficulty of working with calcifying organisms, and the fact that, unlike some other animal-dinoflagellate symbioses, corals readily die upon becoming aposymbiotic (i.e., bleaching) and are consequently not amenable to inoculation and bleaching studies.

Anemones of the genus *Exaiptasia* also engage in symbiosis with Symbiodiniaceae dinoflagellates through mechanisms that are believed to be similar to those characteristic of the most basal coral-Symbiodiniaceae interactions (Lehnert, Burriesci & Pringle, 2012). They are consequently used often as model organisms for studies of coral-dinoflagellate symbioses due to the ease with which they can be maintained in culture in both symbiotic and aposymbiotic states (Weis, 2008; Weis et al., 2008; Sunagawa et al., 2009; Hambleton, Guse & Pringle, 2014); unlike corals, their symbiosis with dinoflagellates is considered facultative, and they are not generally believed to suffer adverse health effects in the aposymbiotic state (provided, presumably, that they are fed regularly).

Typically, the symbiosis between invertebrates and Symbiodiniaceae dinoflagellates is considered mutualistic (Muscatine, 1967; Lewin, 1982). The latter receive CO_2_, phosphate, and ammonium (NH_4_^+^) from animal catabolism (Cook, D’Elia & Muller-Parker, 1988; Lesser & Shick, 1989; Klumpp, Bayne & Hawkins, 1992; Baillie & Yellowlees, 1998; Yellowlees, Rees & Leggat, 2008; Fransolet, Roberty & Plumier, 2012); in turn, the animal hosts receive up to 95% of the dinoflagellate photosynthate (Trench, 1979; Muscatine, 1990), satisfying nearly all of their energetic needs (Fisher, Fitt & Trench, 1985; Falkowski et al., 1993). However, in some cases, these dinoflagellates have been shown to behave more like parasites (Leung & Poulin, 2008). In excavating sponges, a shift to parasitism was observed when irradiance was modified (Fang et al., 2017). In jellyfish, Symbiodiniaceae dinoflagellates become parasitic when acquired via human-induced horizontal transmission, whereas their association with the jellyfish appears mutualistic when the dinoflagellates are acquired naturally via vertical transmission (Sachs & Wilcox, 2006). As another example, in adults of the gastropod *Strombus gigas*, Symbiodiniaceae cells have been observed to be predominantly heterotrophic, bestowing a net cost to the host (Banaszak, Ramos & Goulet, 2013); similar behavior was observed in starved anemones kept in darkness, in which aposymbiotic individuals showed a lower mortality rate compared to symbiotic ones (Steen, 1986). Adverse effects have also been documented in anemones inoculated with heterologous symbionts (Matthews et al., 2017). Furthermore, Symbiodiniaceae dinoflagellates are mixotrophic when free-living (Jeong et al., 2012; Xiang et al., 2013; Xiang et al., 2015; Xiang et al., 2018), and such mixotrophic behavior has been observed *in hospite* (with the algae actually *importing* carbon from their hosts (Dunn et al., 2012).

Most members of the Apicomplexa, a sister group of dinoflagellates, are parasites (Coats, 1999; Lesser, Stat & Gates, 2013); however, the photosynthetic apicomplexan *Chromera velia* (Oborník & Lukeš, 2015) is allegedly capable of symbiotic interaction with corals (Venuleo, Práŝil & Giordano, 2017). Following the hypotheses put forth previously (e.g., Leung & Poulin, 2008; Wooldridge, 2010; Lesser, Stat & Gates, 2013), we hypothesized that we could directly demonstrate whether the interaction between the Symbiodiniaceae dinoflagellate *Breviolum minutum* and *Exaiptasia pallida* indeed shifts away from mutualism under certain environmental conditions. Specifically, we hypothesized that, by limiting food intake, we could change the behavior of *Breviolum* spp. *in hospite* and consequently alter the physiology of their anemone hosts over a multi-week timescale.

## Materials and Methods

### Anemone husbandry, dinoflagellate culture, and anemone inoculation

The sea anemones (*E. pallida*) used in this study were obtained from a single clonal line, PT-1, which was originally isolated from the husbandry center of the National Museum of Marine Biology and Aquarium (NMMBA; Peng et al., 2012). The aposymbiotic anemones were prepared via repeatedly cold shocking individuals until they were completely bleached (*sensu* Muscatine, Grossman & Doino, 1991), and the resulting aposymbiotic anemones were maintained in the dark and fed freshly hatched *Artemia* sp. nauplii weekly for several years. In order to confirm that there were no dinoflagellate cells within these anemones, juveniles (∼2–3 mm in height) were cultured under a 12-h light (40 µmol photons m^−2^ s^−1^): 12-h dark photoperiod at 25 ° C for two weeks. This period allowed residual dinoflagellate cells, if any, to replicate within the anemones, and anemones were examined for presence of dinoflagellates under a fluorescence stereomicroscope (AxioCam SteREO Discovery V8; Zeiss, Germany).

**Figure 1:**
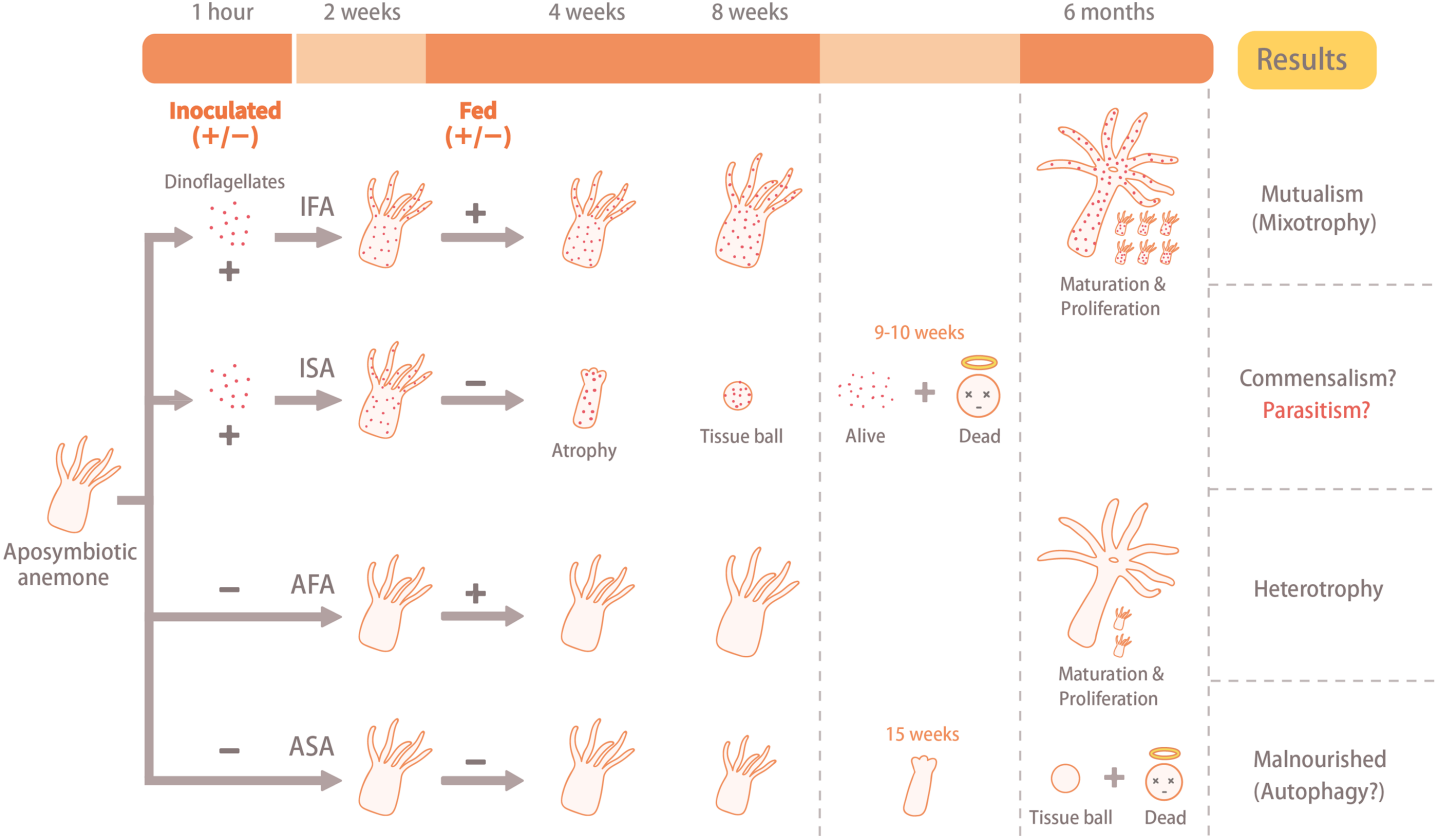
Illustration of the experimental design and results. The aposymbiotic anemones (*Exaiptasia pallida*) were divided into four groups: (1) aposymbiotic and starved anemones (ASA), (2) aposymbiotic and fed anemones (AFA), (3) inoculated and starved anemones (ISA), and (4) inoculated and fed anemones (IFA). Anemones of the IFA and ISA groups were inoculated with dinoflagellates (*Breviolum minutum*; ITS2 type B1) for 1 hour, and cell numbers, densities, and *in hospite* location of dinoflagellates were tracked over eight weeks. In both cases, the circular faces with halos (“dead”) refer to anemones only, and not dinoflagellate cells.

Upon verifying the absence of dinoflagellates, anemones were further cultured in seawater that had been piped in from offshore (N22 03 00.08, E120 41 42.88) and filtered; filtered seawater (FSW) was changed daily. These experimental organisms were then divided into four groups of 12 individuals: (1) aposymbiotic and starved anemones (ASA), (2) aposymbiotic and fed anemones (AFA), (3) inoculated and starved anemones (ISA), and (4) inoculated and fed anemones (IFA) (Fig. 1). The 12 anemones from each treatment were cultured separately in two 6-well cell culture plates (1 anemone per well). Anemones of the IFA and ISA groups were inoculated with the monoclonal homologous dinoflagellate *B. minutum* (ITS2 type B1; strain SBM2), which was originally isolated from an *Exaiptasia* anemone and had been cultured in the laboratory according to Peng et al. (2012) for several years in Guillard’s (f/2) media (without silica, cat. G0154, Sigma-Aldrich, USA) containing antibiotics (10 mg ml^−1^ streptomycin and 10 units ml^−1^ penicillin; cat. 15140-122, Gibco, USA) at 25 °C under the same photoperiod described above. For inoculation trials, we followed a previously established method to prepare symbiotic anemones and confirmed their successful uptake of (and later colonization by) dinoflagellates (Chen et al., 2016). Briefly, the cultured dinoflagellates in the early stationary phase were collected via centrifugation (800 *xg* for 5 min), re-suspended and diluted with 0.22-µm-FSW, and then 15 ml of the dinoflagellate cell solution (3 ×10^5^ cells ml^−1^) were co-cultured with 50 aposymbiotic anemones in a sterile petri dish (90 × 15 mm).

After 1 hr of co-culture, the anemones were washed twice with FSW to remove any dinoflagellate cells that were not engulfed. The inoculated anemones were observed under the fluorescence stereomicroscope to ensure that dinoflagellates had been uptaken, and >90% of the anemones were successfully inoculated; only these were used in experiments. Consequently, all anemones (including the aposymbiotic controls of the ASA and AFA groups) were maintained in 6-well cell culture plates (1 anemone per well) for further observations. In order to prevent the buildup of bacteria, seawater was changed daily. After two weeks of inoculation, the AFA and IFA groups were fed with *Artemia* sp. nauplii (56 well^−1^) once a week; as this was more than the anemones could consume, uneaten nauplii were removed after 30 min. Replicates of all four treatments were visually monitored, and growth and pedal disk measurements (described below) were made weekly.

### Assessment of inoculation, anemone growth, and asexual reproduction

The cell numbers, densities, and *in hospite* location of dinoflagellates in five anemones of the IFA and ISA groups were assessed from whole body images of anemones made after 1 hr, 1 week, 2 weeks, 4 weeks, 6 weeks, and 8 weeks. Anemones were anesthetized in 3.5 and 7% MgCl_2_ in FSW. After incubation (5 min each), the samples were moved to a clean dish with a drop of 7% MgCl_2_ in FSW and imaged under the fluorescence stereomicroscope. The dinoflagellate cell number and the host tissue area were measured by ImageJ (National Institutes of Health, USA) as follows. For measuring the number of dinoflagellate cells within each sampled anemone, the total area of red fluorescence (indicative of the total area comprised by uptaken dinoflagellates) was measured by ImageJ and then divided by 33.064 µm^2^ (the average area of a single dinoflagellate cell; Pasaribu et al., 2015). To obtain the dinoflagellate density within the anemone, the number of dinoflagellate cells was divided by the area of the whole anemone (also measured with Image J). A step-by-step image analysis protocol has been provided (supplemental protocol), though it is worth noting here that there existed the inherent risk of under-reporting actual *in hospite* endosymbiont densities on account of having imaged in only a single focal plane. Data were presented as per anemone and per mm^2^ (Fig. S1).

Growth of anemones was derived from images of pedal disks taken under an inverted microscope (IX70, Olympus, Japan). The measurements of pedal disk size were made with ImageJ. When an anemone of the ISA group formed a “tissue ball” (see below.), its pedal disk size was still measured if part of the pedal disk was still attached to the dish; however, if the pedal disk had disintegrated, that anemone was not analyzed. After the 8-week experiment, anemones of the IFA and AFA groups were subsequently cultured for another six months under the same conditions, and the total number of lacerates produced by each group was observed to further understand the effect of symbiosis on the anemones’ asexual reproduction (as inferred from pedal laceration).

### Transmission electron microscopy (TEM)

Three specimens from the ISA group that had formed tissue balls after prolonged starvation were prepared according to Peng et al. (2011). To compare the difference between disintegrated (tissue ball) and healthy specimens, tentacles of a presumably healthy symbiotic anemone were prepared as a control. Briefly, specimens were fixed in 2.5% glutaraldehyde/2% paraformaldehyde in 100 mM sodium phosphate containing 5% sucrose (pH 7.3) for 2.5 hr at 4 °C, then washed with 100 mM sodium phosphate at 4 °C. They were next post-fixed in 1% OsO_4_ in 50 mM sodium phosphate (pH 7.3) for 1 hr at 4 °C. The specimens were washed with water, dehydrated with increasing ethanol concentrations (50, 70, 80, 90, 95, and 100%), and embedded in Spurr’s resin (Electron Microscopy Sciences, USA). Unfortunately, because tissues balls were so small and difficult to process without disrupting their structure, only one was successfully embedded and sectioned. The embedded specimen blocks were pre-sectioned with an ultramicrotome (Leica Ultracut R) to a thickness of around 200–1000 nm. When gastrodermal tissues appeared in the serial sections (as indicated by intact dinoflagellate organelles under the microscope), the blocks were further sectioned to 70 nm. These 70-nm sections were post-stained in 2.5% uranyl acetate in methanol and 0.4% lead citrate, deposited onto copper grids, air-dried, and imaged under a JEM-1400 transmission electron microscope (JEOL, Japan).

### Statistical analyses

Two-way, repeated-measures ANOVAs were carried out with JMP^®^ (ver. 14) to determine the effects of inoculation status (inoculated vs. aposymbiotic), feeding status (fed vs. sampling time, and their interaction on sea anemone pedal disk size (a proxy for growth), and Tukey’s *post-hoc* tests were conducted with JMP to determine individual mean differences. One-way repeated measures ANOVAs were used to determine the effects of feeding status and time on dinoflagellate cell densities for IFA and ISA only. Contingency table-based chi-squared (*X*^2^) tests were used to analyze the proportional data (likelihood of tissue ball formation and mortality) across treatments and over time. In another test, these proportional data were pooled across the eight sampling times, and the effect of each main experimental factor (feeding status and symbiotic status) was tested individually. *X*^2^ tests were also performed individually across treatments within each sampling time. Given that the majority of samples did not form tissues balls, the resulting Hessian matrix suggested quasi-complete separation of the data; this invalidated the use of the more conservative nominal logistic regression and generalized linear model approaches for analyzing binomial data (tissue ball formation: yes vs. no or mortality: yes vs. no).

## Results

**Figure 2:**
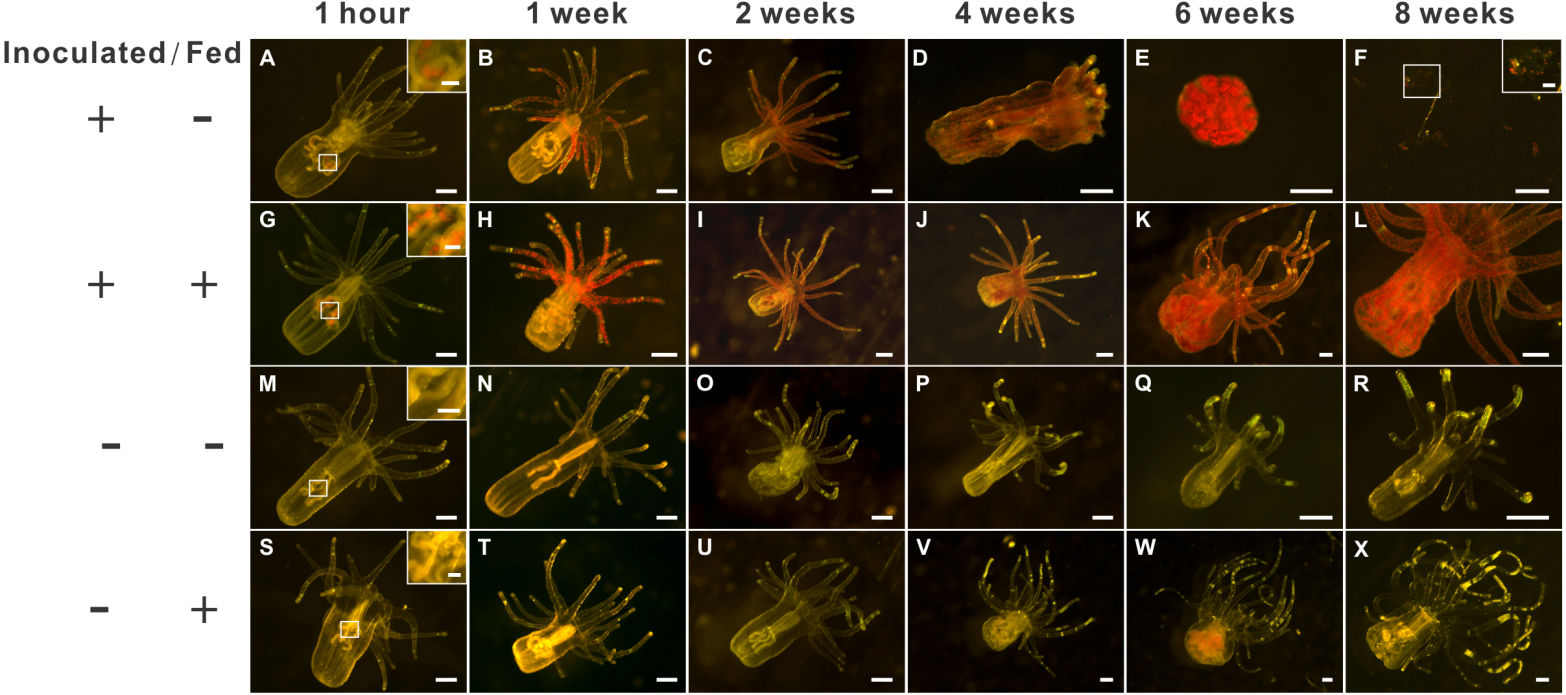
The morphology of aposymbiotic anemones, as well as those inoculated (symbiotic) with dinoflagellates (*Breviolum* spp.), under starved or fed conditions over an eight-week period. The first (A–F), second (G– L), third (M–R), and fourth (S–X) rows depict representative anesthetized anemone samples of the “inoculated-starved” (ISA; +/ −), “inoculated-fed” (IFA; +/ +), “aposymbiotic-starved” (ASA; −/ −), and “aposymbiotic-fed” (AFA; −/ +) treatments, respectively, and scale bars represent 200 µm except for in the 1-hour and F insets (50 µm). The weak red autofluorescence in Fig. 1W represents undigested food (*Artemia* nauplii), not dinoflagellate cells.

*E. pallida* anemones of the IFA and ISA groups were successfully inoculated with the monoclonal, cultured, and homologous *Breviolum* dinoflagellates (SBM2). One-hour post-inoculation, the *Breviolum* cells were observed in the gastric cavity of each anemone (Fig. 2A, 2G), and the dinoflagellate cells were initially concentrated in the column (with only a few present in the tentacles). After 1 to 2 weeks, the dinoflagellate cells proliferated (Fig. 2B–2C, 2H–2I; Fig. S1A–S1B), and the average dinoflagellate cell numbers were 126, 2,896, and 9,617 cells per anemone after 1 h, 1 week, and 2 weeks in the ISA groups, respectively, and 244, 3,113, and 10,687 cells per anemone, respectively, in the IFA group (Fig. S1A).

Two-weeks post-inoculation, the average dinoflagellate cell density within the anemones increased significantly over earlier observation times, nearly saturating the anemone tissues; the densities were similar in the ISA and IFA groups (17.6 ×10^3^ cells mm^−2^ and 16.4 ×10^3^ cells mm^−2^, respectively; Fig. S1B). After confirming that both groups of anemones were colonized to a similar degree, we began to feed those of the IFA group. The aposymbiotic control groups, AFA and ASA, remained symbiont-free for the entire duration of the study (see Fig. 2M–2R and 2S–2X for representative images.) From the 4th week onwards, the endosymbiont cell counts differences between IFA and ISA became more pronounced; this is evident from both images of representative anemones (Fig. 2D) and cell counts data per whole anemone (Fig. S1A). Curiously, when accounting for the reduced size of the ISA (normalizing to anemone surface area), the endosymbiont density, in fact, remained similar to that of IFA over time (Fig. S1B).

**Figure 3:**
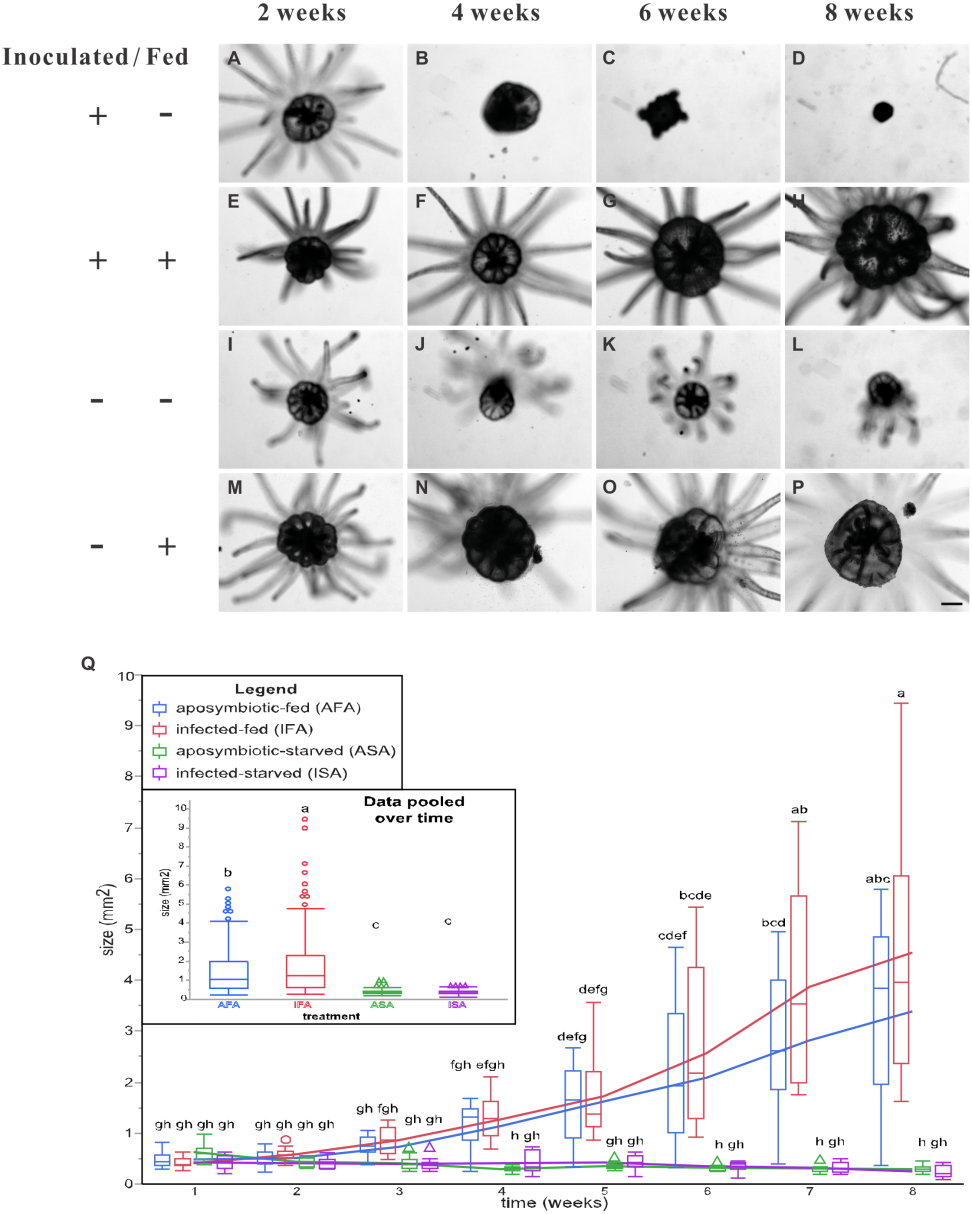
The pedal disk sizes of *Breviolum*-inoculated (symbiotic) and aposymbiotic anemones under starved or fed conditions over an eight-week period. Images of all anemones were captured every two weeks with an inverted microscope to record their growth (A–P), and representative samples have been shown (scale bar: 400 µm). By the 6th week of inoculation, the inoculated and starved anemones (ISA) no longer displayed normal morphology. In Q, pedal disk area data (as normal quantile plots) have been plotted over time for the four treatment groups (*n* = 12), and lowercase letters represent Tukey’s *post-hoc* differences (*p* < 0.05). In an inset, the pooled means across the eight sampling times have been shown, and lowercase letters above error bars reflect Tukey’s *post-hoc* differences. Please note that the pedal disk areas of the fed groups (AFA and IFA) were significantly higher than their respective starved group (ASA and ISA) from the 6th week onwards. Although IFA was larger than AFA upon pooling the data (inset), no within-time *post-hoc* differences were uncovered.

Overall, the two fed groups (IFA and AFA) grew to larger sizes, and feeding had a significant effect in the two-way, repeated measures ANOVA (Table 1); specifically, pedal disk analysis (Fig. 3) confirmed that the fed groups were appreciably larger from the 4th week post-inoculation onwards, and such differences were statistically significant by the 6th week (Fig. 3Q). By the 8th week, anemones of the IFA and AFA groups were ∼20- and ∼10-fold larger, respectively, than the ISA and ASA groups, respectively (Fig. 3Q); interestingly, anemones of the IFA and AFA were similarly sized. For the starved groups (ISA and ASA), the anemones did not grow appreciably over time (Fig. 3Q); in fact, pedal disk areas shrunk from 0.44 mm^2^ at week 1 to 0.22 mm^2^ at week 8 (ISA) and from 0.58 mm^2^ to 0.27 mm^2^, respectively (ASA), though these differences were not statistically significant.

**Table 1:** Two-way, repeated measures ANOVA of the effects of feeding status (fed vs. starved) and symbiotic status (inoculated vs. aposymbiotic) over time on sea anemone size (mm^**2**^**; *n* = 12/each of four treatments)**. In the “comparison” column, lowercase letters in parentheses trailing treatment groups signify Tukey’s *post-hoc* inter-mean differences (*p* < 0.05), and, in two cases (feeding status and time × feeding status), the overall fold changes between the two most extreme values have been shown in parentheses. Since there were 16 interaction groups for time × feeding status, only the two most extreme (maximum and minimum, respectively) values have been list in the cell. Despite the absence of an overall time × feeding × symbiotic status effect, there were numerous *post-hoc* differences between the 32 interaction groups; see Fig. 2B.

**Factor**	**df**	**Exact-*F***	***p***	**comparisons / notes**
Feeding status	1	78.5	<0.001	fed(a)>starved(b) (4.5-fold)
Symbiotic status	1	1.75	NS	NA
Feeding ×symbiotic status	1	1.70	NS	NA
Time	7	16.0	<0.001	8(a), 7(ab), 6(bc), 5(cd), 4(de), 3(de), 2(e), 1(e)
Time ×feeding status	7	24.1	<0.001	fed-8-week (a)>starved-8-week (e) (∼15-fold)
Time × symbiotic status	7	1.74	NS	NA
Time × feeding × symbiotic status	7	0.985	NS	see Fig. 2B

**Notes.**

NA not applicable NS not statistically significant (*p*> 0.01)

**Figure 4:**
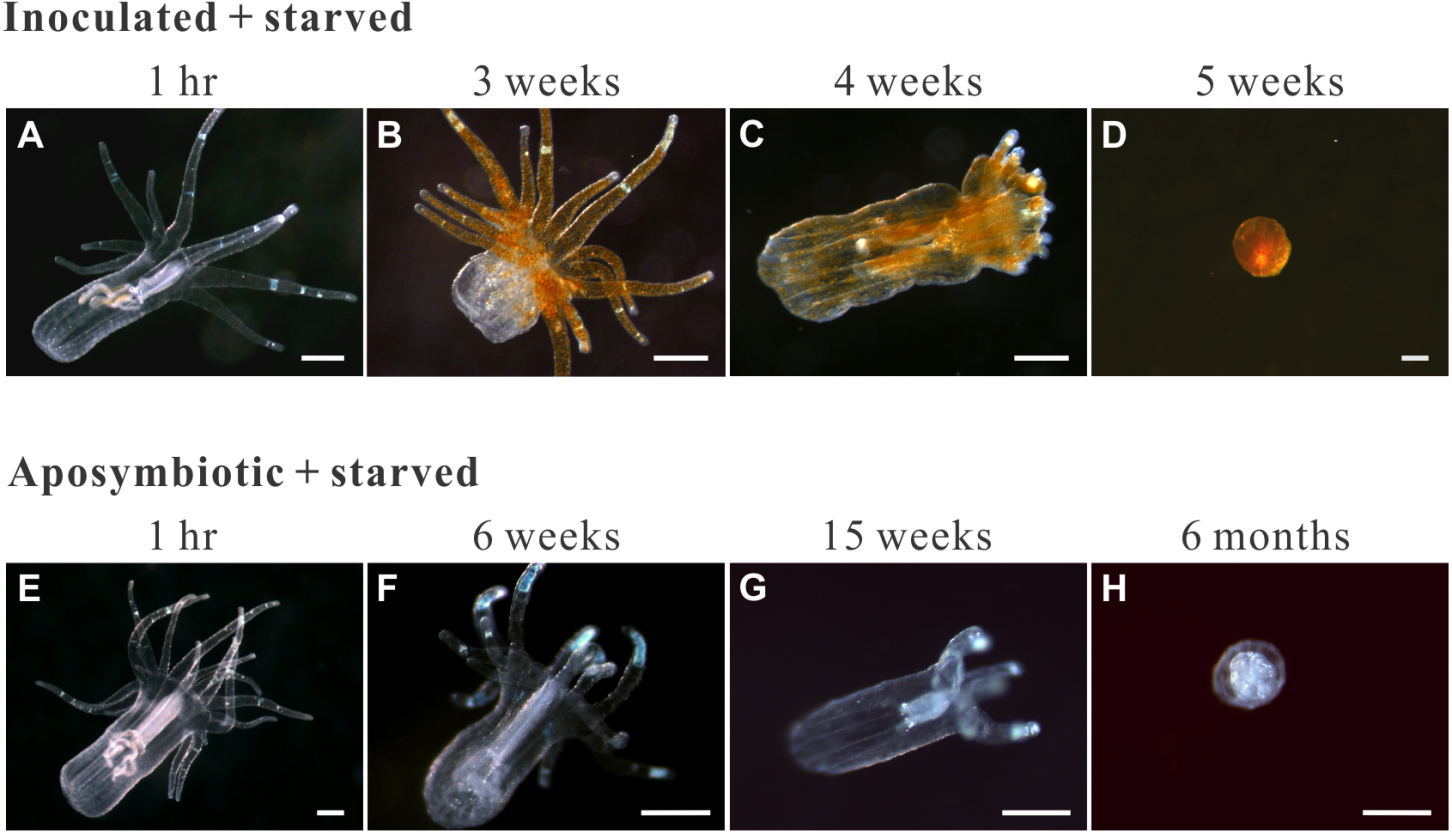
Morphological changes in representative starved anemones. Both inoculated (red-brown dots represent dinoflagellate cells.) and aposymbiotic anemones proceeded through the same four morphological stages (from left to right), albeit typically at different times: classical/healthy anemones (1-hr for both treatment groups), reduced-size individuals (though still in anemone form; 3 and 6 weeks for inoculated-starved [ISA] and aposymbiotic-starved [ASA] anemones , respectively), individuals with reduced tentacle numbers (4 and 15 weeks for ISA and ASA, respectively), and “tissue ball” (5 weeks and 6 months for ISA and ASA, respectively; see Table 2 for details.). All scale bars: 200 µm.

**Figure 5:**
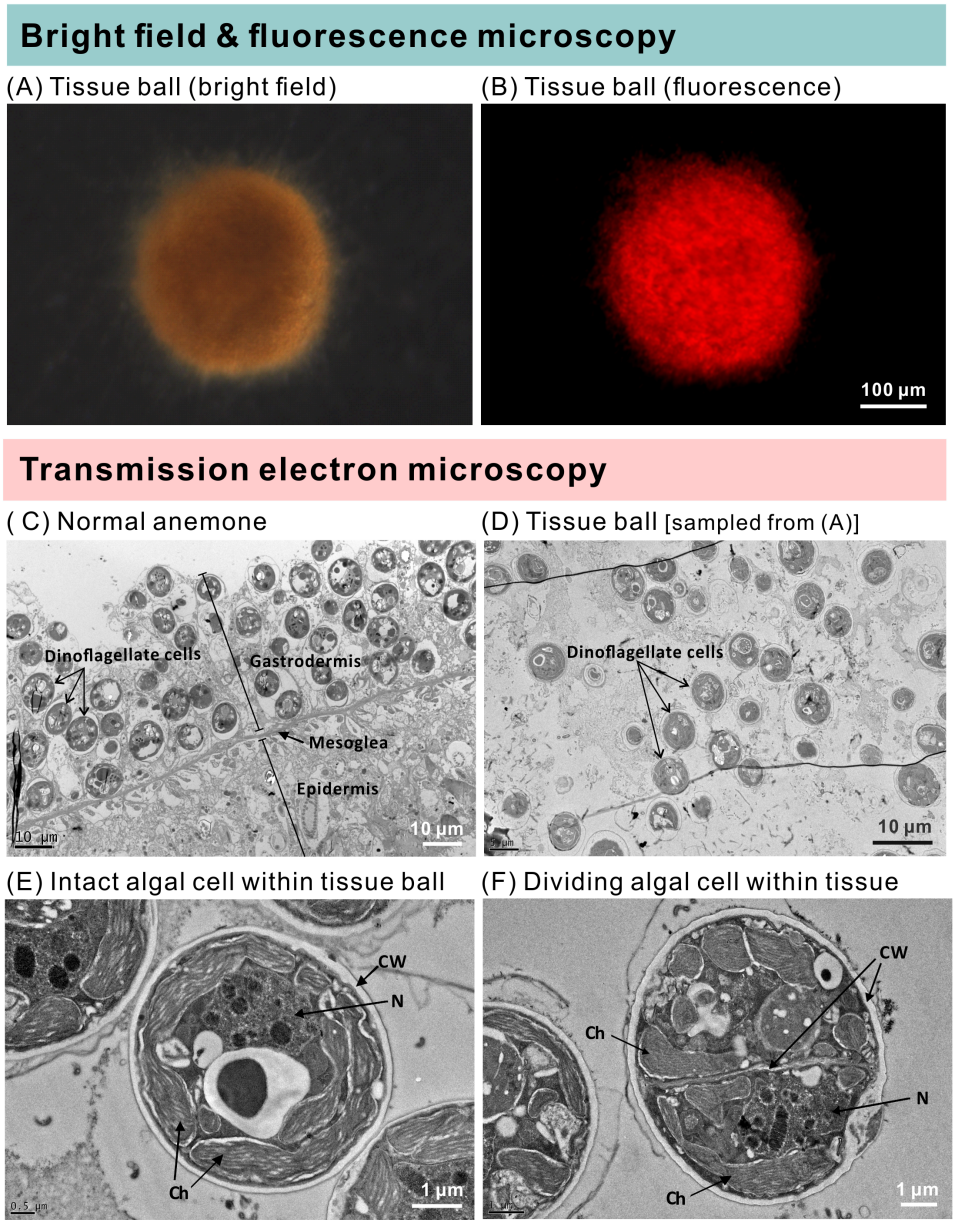
The morphology and ultrastructure of an anemone tissue ball from the inoculated+starved treatment group. (A) A tissue ball imaged under bright field microscopy. (B) The same tissue ball as in (A), though imaged under fluorescence microscopy. (C) Ultrastructure of tentacular tissue sampled from a normal anemone. (D) Within the tissue ball, host cells were disintegrating, but the cellular structures of dinoflagellate cells remained intact. (E) An intact dinoflagellate cell within the tissue ball; the cell wall (CW), chloroplasts (Ch), and nucleus (N) are all evident. (F) A dividing dinoflagellate cell (doublet) within the tissue ball.

The groups differed not only in their growth but also in their external morphology (Fig. 2). While the fed groups showed the typical anemone morphology (Figs. 2G–2L and 2S–2X), the starved ones demonstrated morphological changes, which were especially evident in the ISA group by the 4th week (Fig. 2A–2F); specifically, these individuals were characterized by smaller column sizes and reduced tentacle size and number (Fig. 2D; Fig. 4C). Four to five weeks after inoculation, anemones from the ISA group began to morph into spherical shapes. At this stage, it was still possible to distinguish the oral disk from the mesenteries. These anemones eventually formed tissue balls lacking in recognizable structure (Fig. 2E; Fig. 4D; Figs. 5A–5B); by the 8th week, 100% of the samples in this treatment group had formed tissue balls (Table 2). There was a statistically significant effect of treatment on likelihood of tissue ball formation (Table 2). Furthermore, 25% of the ISA had died by the 8th week. In contrast, anemones of no other treatments formed tissue balls, except for ASA after six months of observation (Figs. 4E–4H). In the tissue balls of the ISA, the host tissues eventually disintegrated (Fig. 5D); however, dinoflagellate cells appeared intact, and cell walls, chloroplasts, nuclei, and chromosomes were observed (Figs. 5E–5F). Even a dividing dinoflagellate cell was observed (Fig. 5F). Moreover, tissues ball did not bleach (Figs. 5A–5B), and the density of symbiotic dinoflagellates in the tissue ball actually increased (Fig. S1B).

**Table 2:** Tissue ball formation and mortality of aposymbiotic and ***Breviolum*-inoculated (symbiotic) anemones that were either starved or fed across an 8-week (w) period**. Values in cells indicate the proportion of anemones (*n* = 12/treatment) that (1) transformed into tissue balls (top half of table) or (2) died (bottom half of table) at each week of the 8-w observation period. There was a statistically significant effect of treatment (symbiotic status x feeding status) on likelihood of tissue ball formation (*X*^2^ = 119, *p* < 0.001) when pooling data across the entire duration of the experiment. When assessing the effects of feeding (fed vs. starved) and symbiotic status (inoculated vs. aposymbiotic) individually for likelihood of forming tissue balls, *X*^2^ values of 56.9 (*p* < 0.001) were calculated for both upon pooling the data over time. In contrast, only symbiotic status significantly affected mortality (*X*^2^ = 5.91, *p* < 0.05), albeit marginally, at the 8-w observation time; feeding status alone did not affect mortality, nor did the interaction of feeding and symbiotic status (*p* > 0.01). However, when considering the mortality data across the entire duration of the experiment, treatment did significantly affect mortality (*X*^2^ = 19.5, *p* < 0.001); specifically, inoculated and starved anemones (ISA) were statistically more likely to die than aposymbiotic anemones (either fed (AFA) or starved (ASA)), and ISA were more likely to die than ASA (*X*^2^ = 11.7, *p* < 0.001). In the “significant effect of treatment” and “significant effect of symbiotic status only” rows, annotations reflect results of within-time *X* ^2^ tests across the four treatment groups.

**Tissue ball formation**	1w	2w	3w	4w	5w	6w	7w	8w
ISA	0	0	0	1/12	6/12	8/12	11/12	12/12
IFA	0	0	0	0	0	0	0	0
ASA	0	0	0	0	0	0	0	0
AFA	0	0	0	0	0	0	0	0
***Significant effect of treatment***					**	***	***	***
**Mortality**								
ISA	0	0	0	0	1/12	1/12	3/12	3/12
IFA	0	0	0	1/12	1/12	1/12	1/12	1/12
ASA	0	0	0	0	0	0	0	0
AFA	0	0	0	0	0	0	0	0
***Significant effect of symbiotic status only***							*	*

**Notes.**

* *p* < 0.01.

** *p* < 0.001.

*** *p* < 0.0001.

IFA inoculated and fed anemones

Fed anemones (both inoculated and aposymbiotic) were further cultured for six months to allow them to (potentially) grow to full size. During this period, asexual reproduction (laceration) was documented, and the aposymbiotic (AFA) and inoculated (IFA) groups produced 9 and 30 lacerates, respectively.

## Discussion

Regularly fed *Exaiptasia*-*Breviolum* holobionts increased in size and exhibited high asexual reproduction rates and low mortality rates (only 1 of 12 fed anemones died.). However, their ability to grow and reproduce cannot be solely attributed to their association with photosynthetic dinoflagellates; symbiotic anemones that were deprived of exogenously supplied food failed to grow (Figs. 1 and 2). Whether this was due to host malnourishment or, as we hypothesize herein, a shift in the behavior of the *Breviolum* populations *in hospite*, remains to be determined. Certainly nutrient labeling approaches (Cui et al., 2019) should be undertaken in the future to determine whether, for instance, *Breviolum* cease to translocate fixed carbon to the host anemones, or even uptake organic nutrients from them, under such starved conditions. Nevertheless, we now explore the potential shift away from mutualistic behavior by the homologous dinoflagellate *B. minutum* upon integration of our data with findings from the primary literature.

**Figure 6:**
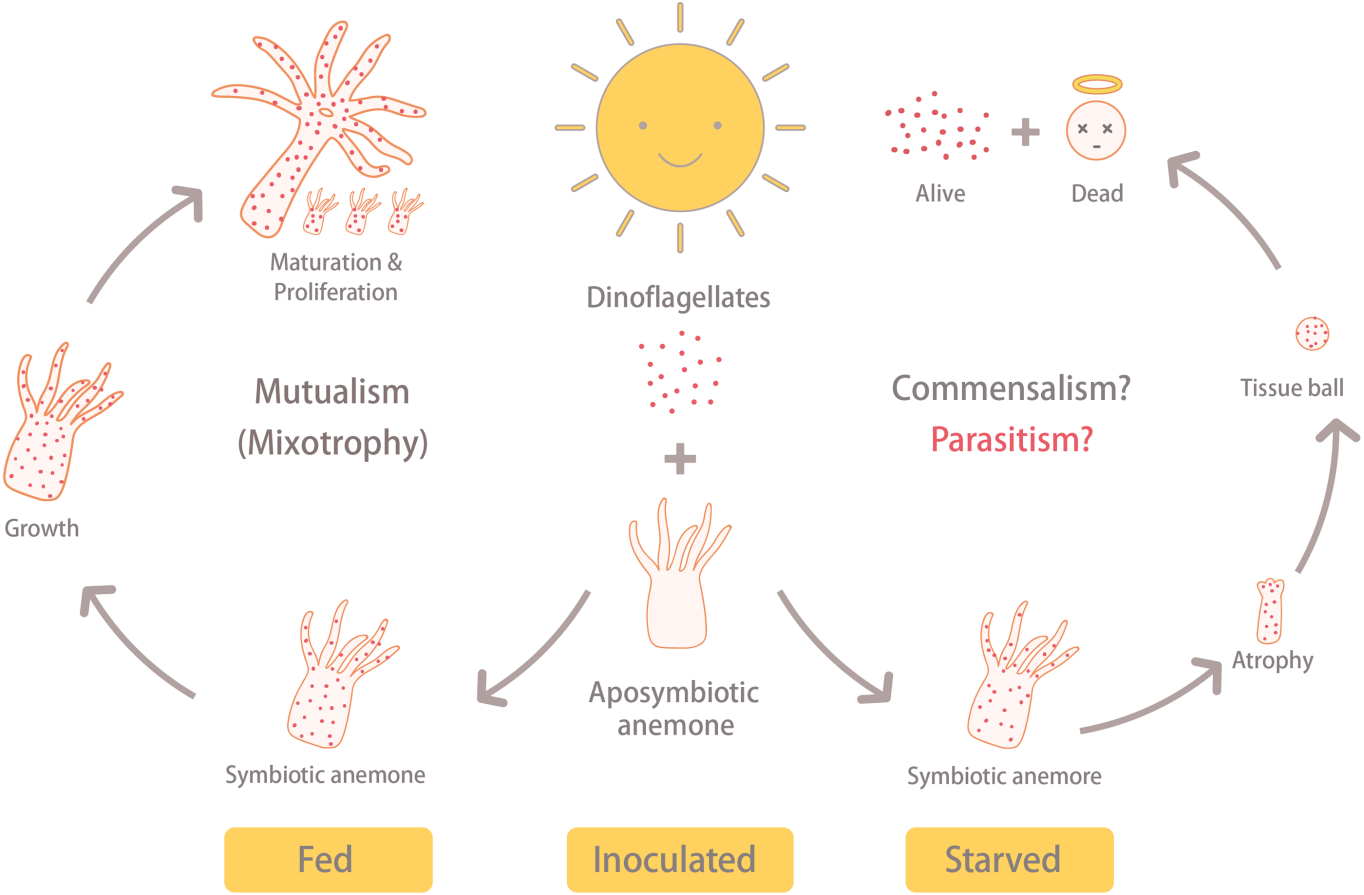
Summary of results. Under the same light/dark cycle, the inoculated and fed (IFA) anemones (i.e., symbiotic) grew, matured, and reproduced asexually via pedal laceration. On the other hand, the inoculated and starved anemones (ISA) shrunk into a tissue ball and disintegrated. Eventually, the ISA hosts died, but the dinoflagellate survived. This reveals the possibility that under stressful conditions, the anemone-dinoflagellate endosymbiosis may shift away from mutualism.

Steen (1986) revealed that Symbiodiniaceae dinoflagellates can survive extended periods of time in darkness due to their capacity to obtain organic nutrients from their anemone hosts. Moreover, the same study demonstrated that symbiotic anemones in complete darkness had a higher mortality rate than aposymbiotic ones. Baker et al. (2018) obtained similar results from working with corals (*Orbicella faveolata*); specifically, they found that seawater warming, even in non-limiting food conditions, induced the symbionts to (1) release less photosynthate and (2) increase the uptake of resources from the host. These studies suggest that Symbiodiniaceae heterotrophy *in hospite* can impose a fatal metabolic burden on their anthozoan hosts, which could explain why our symbiotic, albeit starved, anemones failed to grow and instead formed tissue balls (or even died) in many cases. The fact that aposymbiotic anemones that were fed, in contrast, continued to grow may signify that the dinoflagellates in the starved anemones were indeed compromising the health of their hosts. It is worth noting here that the starved-symbiotic anemones (ISA) did not bleach. Moreover, when the anemone tissues disintegrated, the symbiotic dinoflagellates still displayed normal cellular ultrastructure and were capable of dividing; the fact that they were survived and proliferated in disintegrating host tissue is further evidence for either commensal, or maybe even parasitic, behavior (Fig. 6).

There are several reports that have explored the potential for nitrogen limitation to be a means of inducing symbiont lipid biogenesis/accumulation (Peng et al., 2012) and density control (Cui et al., 2019) in *Exaiptasia* anemones. Under nitrogen-limited or deprived conditions, the dinoflagellates regularly accumulate lipid bodies (LBs), regardless of whether they are free-living or *in hospite* (Jiang, Pasaribu & Chen, 2014; Weng et al., 2014). In this study, there was no LB accumulation within dinoflagellate cells of disintegrating host anemones (Figs. 5D–5F). Instead, the morphology of these dinoflagellate cells was similar to the nutrient-sufficient control specimens of the afore-cited works. Given that dinoflagellate cells did not show signs of nitrogen starvation, but instead continued to grow under these conditions, it is possible that they were acquiring nitrogen from the dying host cells. Nutrient labeling approaches (*sensu* Cui et al., 2019) could surely aid in verifying this.

In *Hydra*, an asymbiotic cnidarian, starvation-induced autophagy is a critical survival mechanism under food-deprived conditions. Upon degrading their own nonessential cells/tissues to obtain amino acids, lipids, and other nutrients, their tentacles and overall body sizes shrink, and they cease undergoing asexual reproduction (Chera et al., 2009). In this study, the ASA and ISA groups behaved similarly to the starved *Hydra*, as their tentacle and body size decreased gradually. Since autophagy is a highly conserved process in all eukaryotes (Parzych & Klionsky, 2014), we suggest it may play a critical role in regulating the cellular stress responses and metabolic modulation of the starved anemone. Our findings might also suggest that the symbiotic dinoflagellate cells appear to accelerate the self-degrading/recycling mechanisms of their host anemones, or even benefit from this process, because the anemones of the ISA group only survived for 2 months, while the ASA group survived for 6 months.

In addition to the other instances of mutualism-to-parasitism shifts mentioned in the Introduction, it is worth noting that mutualism is typically thought to have evolved from parasitism, at least in the case of Symbiodiniaceae dinoflagellates (Stat, Morris & Gates, 2008). This (point #1), along with the aforementioned relationship between dinoflagellates and parasitic apicomplexans (point #2; Lesser, Stat & Gates, 2013) and the microalgal capacity for heterotrophy (point #3; Jeong et al., 2012; Xiang et al., 2015; Xiang et al., 2018) all lend credence to our hypothesis that the delicate metabolic and physiological equilibrium between invertebrates and intracellular dinoflagellates may transition away from being one of mutual benefit to both partners when certain environmental conditions deteriorate. Further work must be undertaken, though, in order to know whether the dinoflagellates within the starved anemones are also malnourished or if they are truly acting as parasites (i.e., absorbing nutrients from their dying hosts). Given that the marine regions in which coral reefs are found tend to be oligotrophic (and therefore of limited potential food supply), the answer to this question may have profound implications for the future survival of coral reef ecosystems.

## Conclusions

Given that anemones that were inoculated with dinoflagellate endosymbionts and consequently starved failed to grow, and finally disintegrated into tissue balls, whereas those inoculated anemones that were fed instead continued to grow, we surmise that, under stressful conditions, the anemone-dinoflagellate endosymbiosis may shift away from one that benefits both partners. Whether this is due to truly parasitic behavior of the endosymbionts, rather than simply commensalism coupled with host starvation, remains to be determined and should be the focus of future studies. These findings and raised hypothesis, therefore, contribute to the field and how we view symbiosis across a spectrum of mutualistic and parasitic relationships.

##  Supplemental Information

10.7717/peerj.9745/supp-1 Data S1 Raw data

10.7717/peerj.9745/supp-2 Supplemental Information 1 Supplemental protocol

10.7717/peerj.9745/supp-3 Figure S1 Dinoflagellate cell counts per anemone (A) and normalized to surface area (mm^2^; (B) for a subset of five individuals from the inoculated and fed (IFA; red) and inoculated and starved (ISA; purple) treatments (as normal quantile plots) The results of the repeated measures ANOVAs (feeding status over time) have been presented in an inset in (A)(log and untransformed data for cells/anemone and cells/ mm^2^, respectively). Although there was not a statistically significant interaction effect for the cells/anemone data (A), Tukey’s *post-hoc* tests nevertheless revealed intra-time treatment differences at the 6- and 8-week (w) sampling times (*p* < 0.05; denoted by asterisks).

## Additional Information and Declarations

### Data Availability

The following information was supplied regarding data availability: All raw data associated with the article are available as Supplemental Files.
