# Supplementary material for: A shift away from mutualism under food-deprived conditions in an anemone-dinoflagellate association
Source: PeerJ. 2020 Oct 28;8:e9745. doi: 10.7717/peerj.9745 (PMC7602683; doi:10.7717/peerj.9745)
Supplement: Supplemental Information 1 [file peerj-08-9745-s002.docx]

**Supplemental protocol**

**Anemone culture and imaging**

Tiny *Exaiptasia* anemones (~2 mm in length) were maintained in 6-well cell culture plates (1 anemone per well) in which filtered seawater (FSW) was changed daily. After anesthetizing in 3.5 and 7% MgCl_2_ in FSW (5 min each), each anemone was moved to a clean dish with a drop of 7% MgCl_2_ in FSW and imaged under an AxioCam SteREO Discovery V8 fluorescence microscope (Zeiss, Germany). Then, each anemone was washed 3-4 times with FSW (3-5 minutes/each) and returned to its cell culture plate.

**Image analysis**

The dinoflagellate cell number and host anemone tissue area were measured by ImageJ (National Institutes of Health, USA) as follows.

1. The brightness of the image taken from the fluorescence microscope may require adjustments of brightness and contrast beforehand. The procedure is as follows:
2. Open Image J and choose the picture for analysis by clicking File → Open → select picture (TIFF or JPEG format).
3. Click on the Image → Adjust →Brightness/Contrast and note the adjustment scale (Fig. 1).


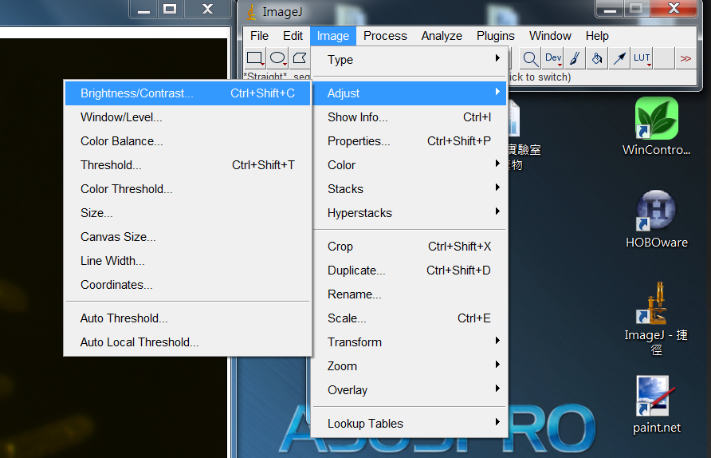

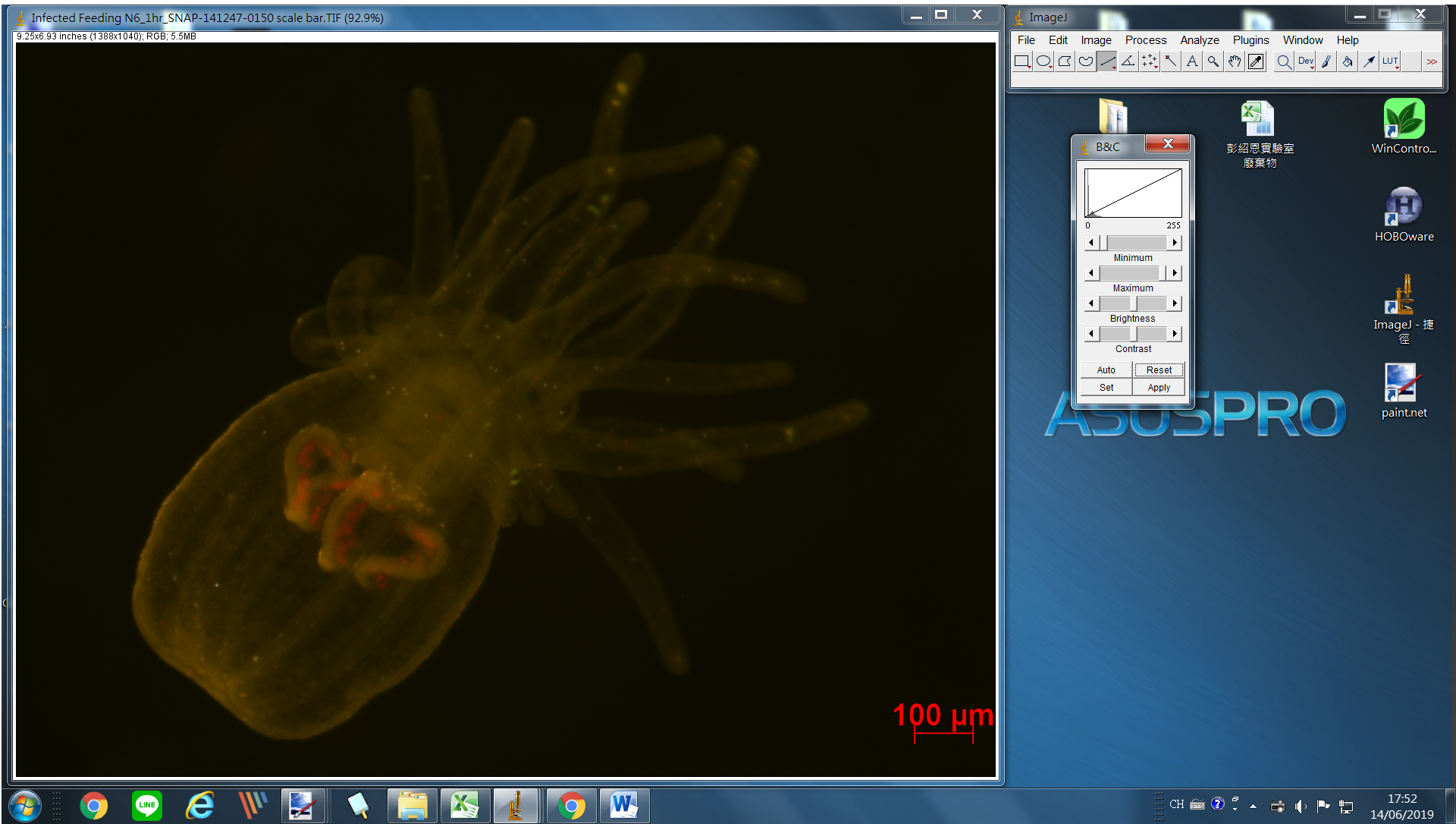


**Figure 1.** The Brightness/Contrast adjustment scale.

1. Adjusting the Brightness/Contrast of the image (Fig. 2).


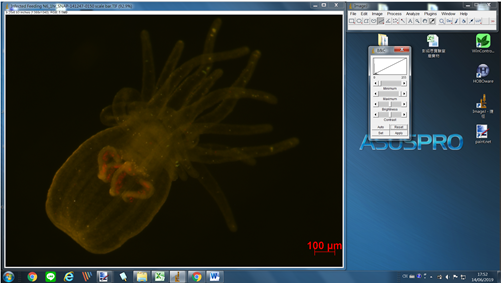

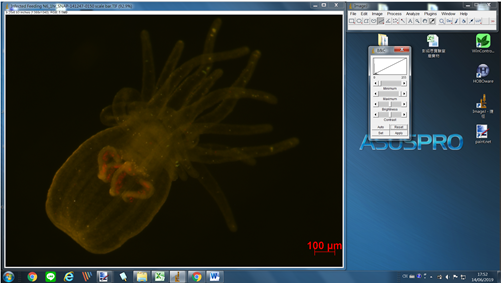


**Before**


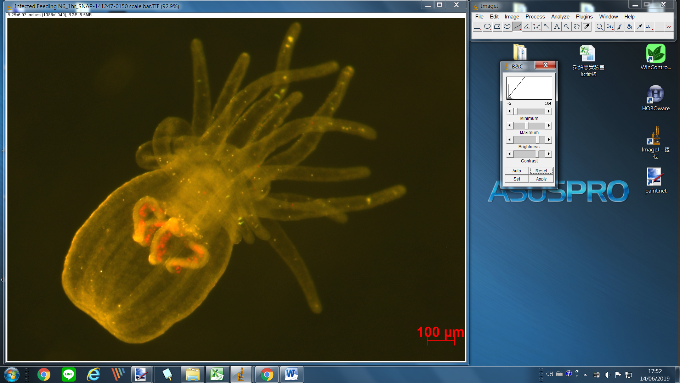

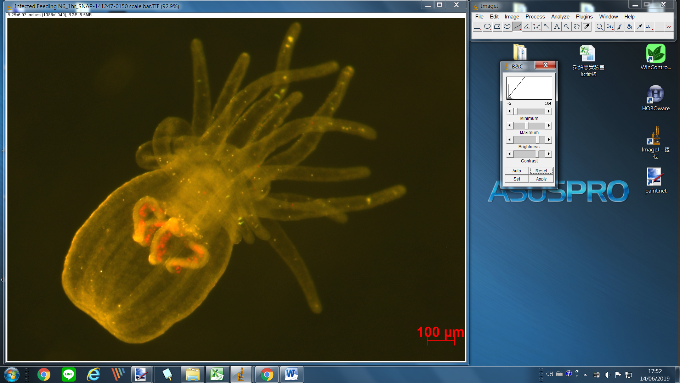


**After**

**Figure 2.** The image before and after brightness/contrast adjustment.

1. Set the scale bar for size measurements.
2.
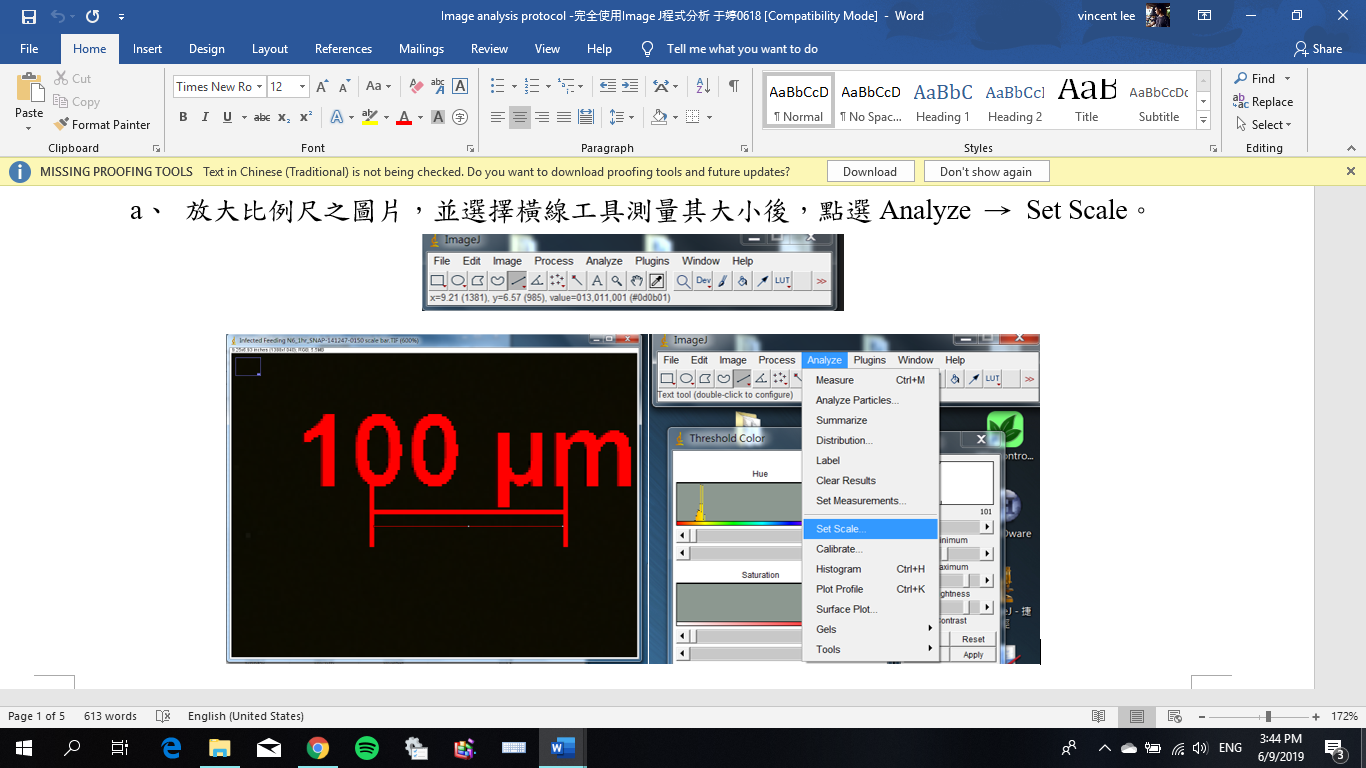
Zoom in to the scale bar on the picture. Click on the straight-line drawing tool and trace the entire length of the scale bar. Click on the toolbar ribbon Analyze → Set Scale (Fig. 3)


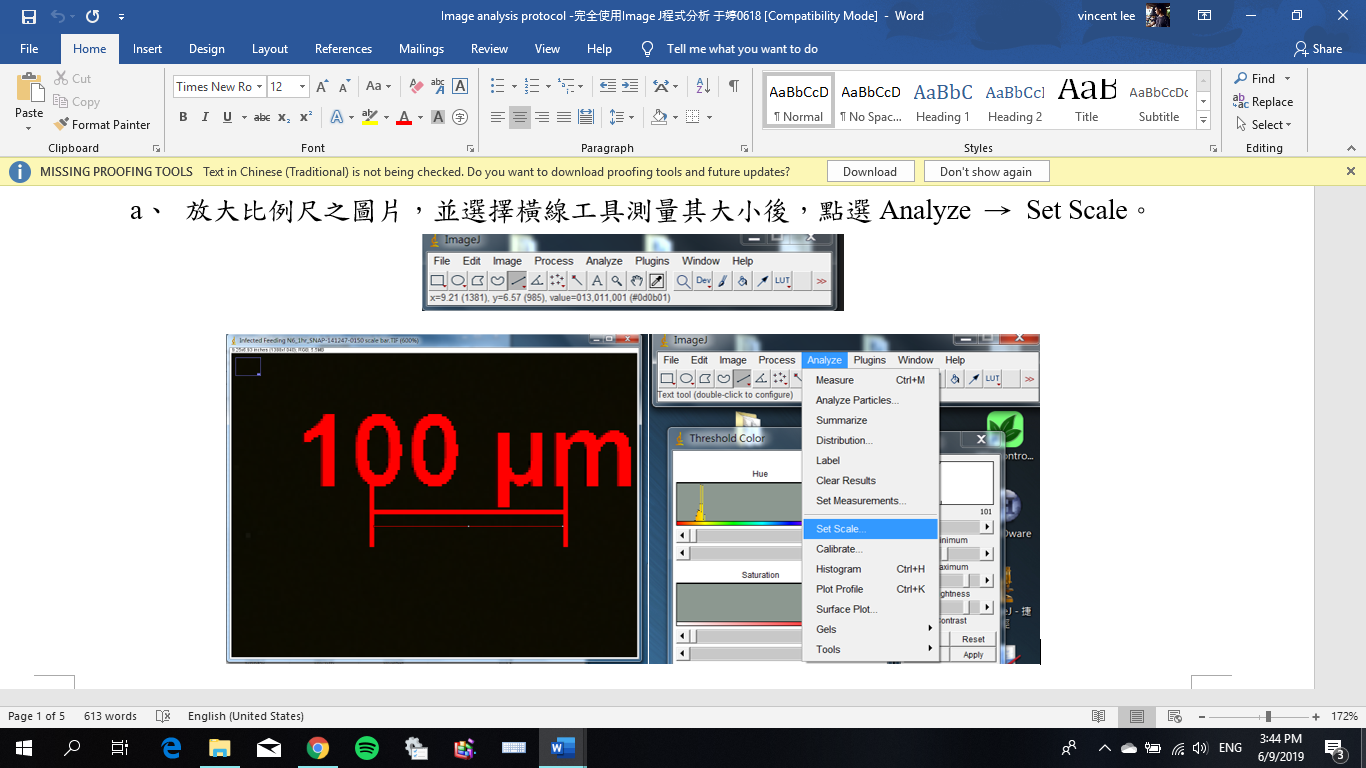


**Figure 3.** Selecting and setting the scale bar.

1. Click on “Known Distance” in the pop-up notification after clicking “Set Scale” and enter the value from the scale bar. Make sure to change the “Unit of length” to match that of the image. Click “OK” (Fig. 4).


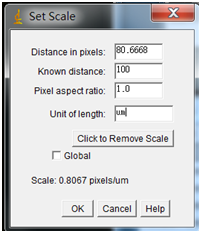

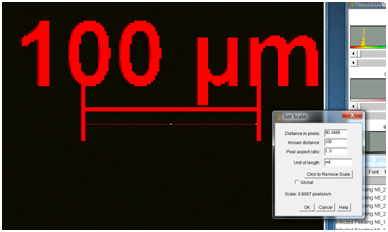


**Figure 4.** Setting the scale.

1. Image analysis – total area of dinoflagellate cells
2.
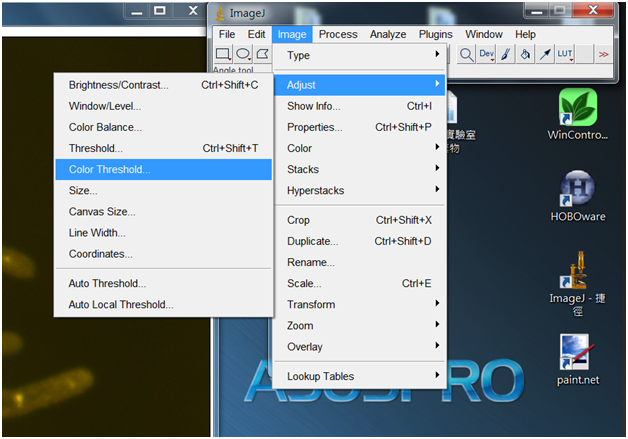
Click on Image → Adjust → Color Threshold to differentiate the dinoflagellate cells in the photo through color contrast (Fig. 5).

**Figure 5.** Selection of “Color Threshold” on the toolbar.

1.
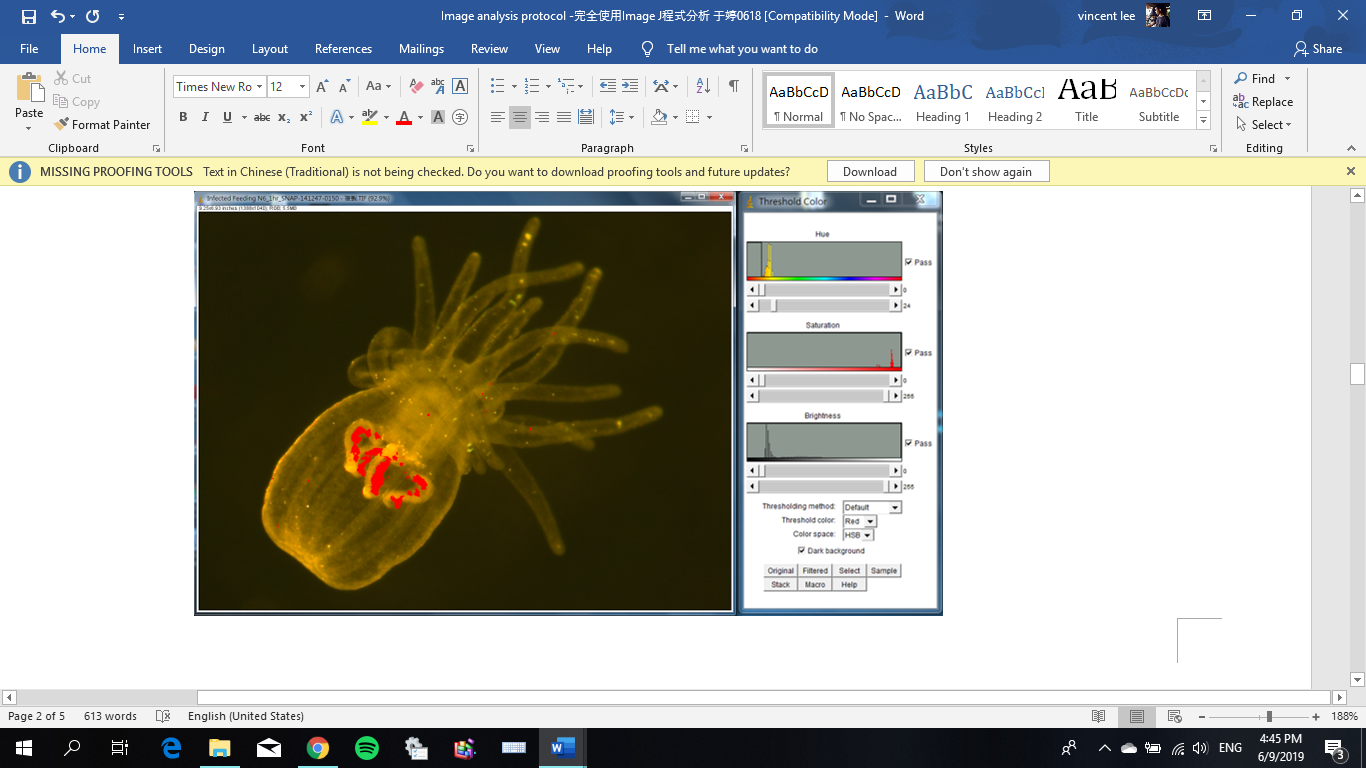

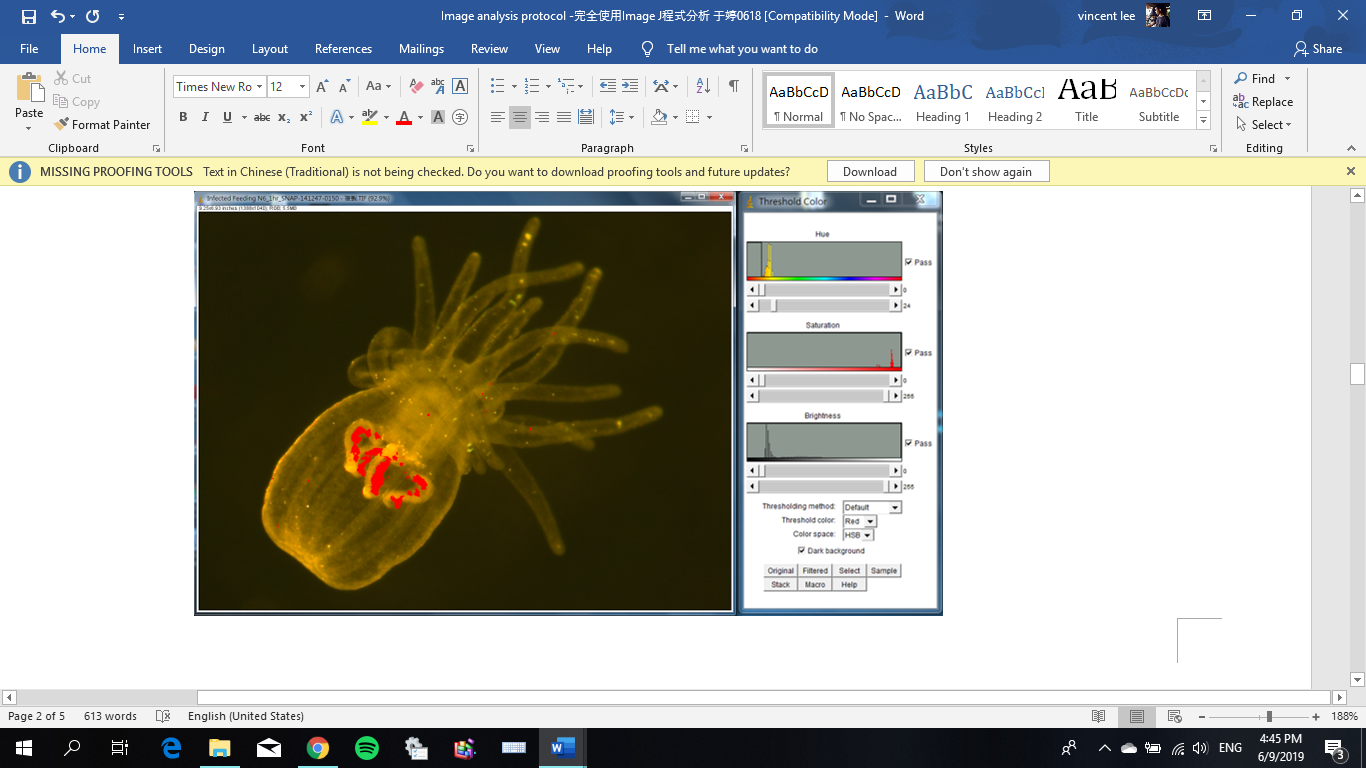
Choose “HSB mode” in “Color Space,” set the “Threshold color” to red. Then, drag the “Saturation” and Brightness” icons on the right side (maximum values) to adjust the area of red color for covering the dinoflagellates cell in the anemone (Fig. 6). Then click “Select” after confirming the area of dinoflagellate coverage in the sea anemone.

**Figure 6.** Selection of the area coverage for dinoflagellates in a sea anemone.

1.
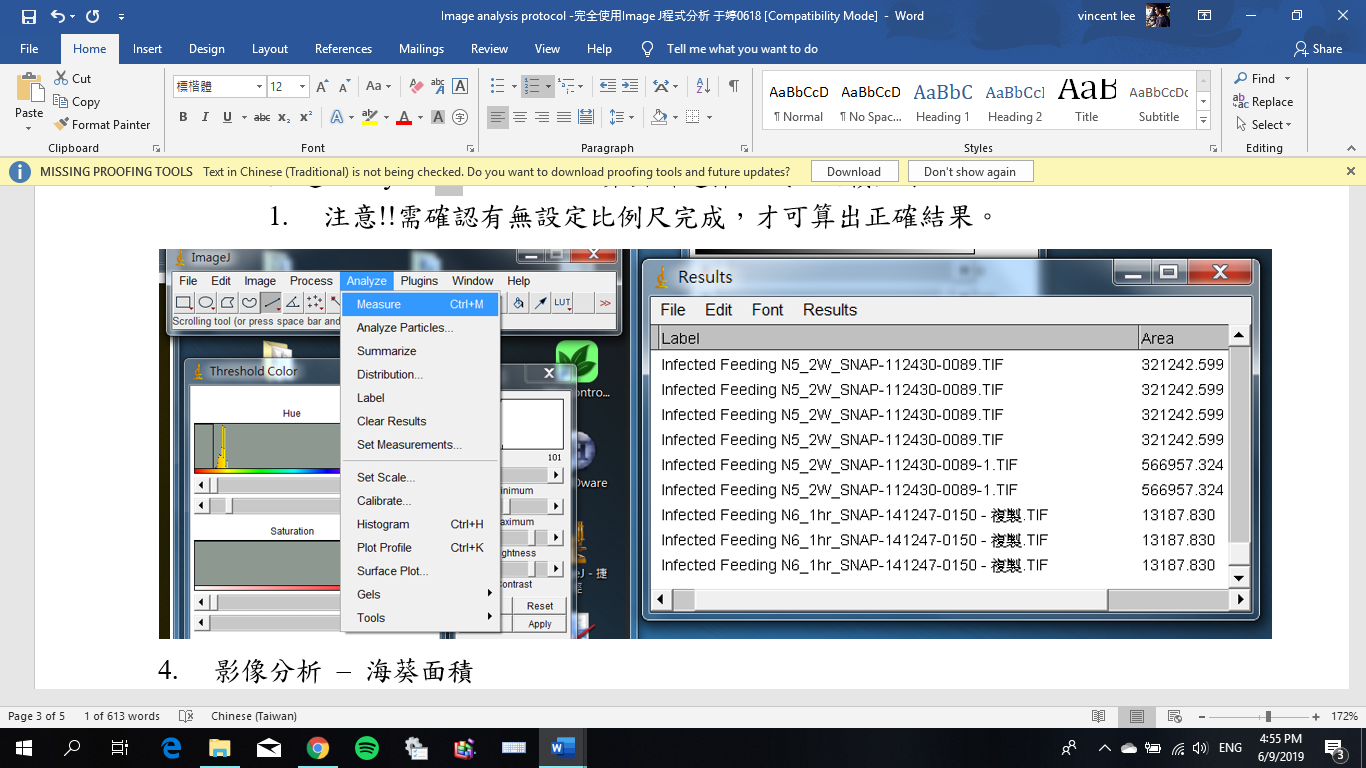
Click Analyze → Measure to calculate the area in the image fulfilling the set criteria (Fig. 7). **Note**: be sure to set the scale bar first, as described above.

**Figure 7.** Measurement of the area occupied by dinoflagellates.

**【Example】 the total area of all dinoflagellate cells is 13,187.83 μm^2^ .**

1. Image analysis – area of anemone
2. Follow the steps as for evaluating the area of dinoflagellates, though instead modify the “Brightness” and “Saturation” parameters to where the entire anemone is featured in red (Fig. 8).


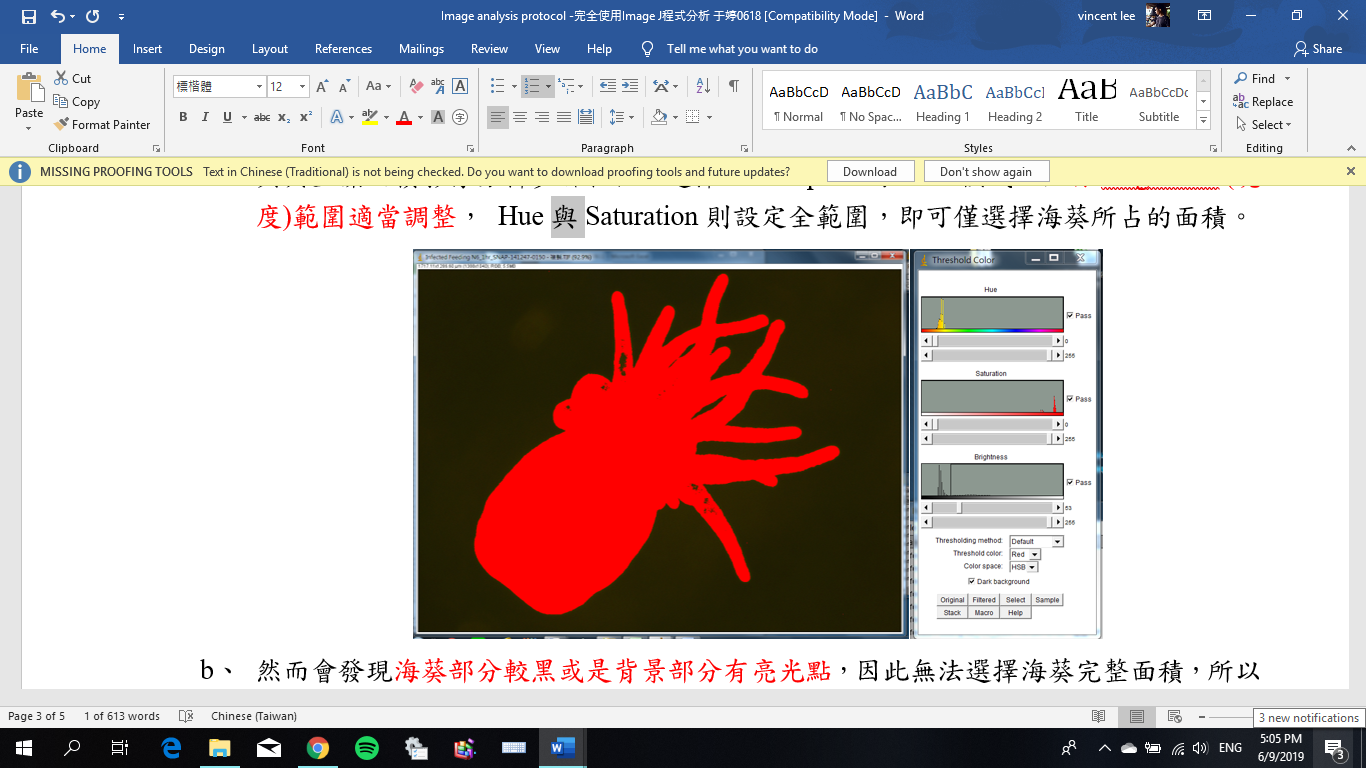

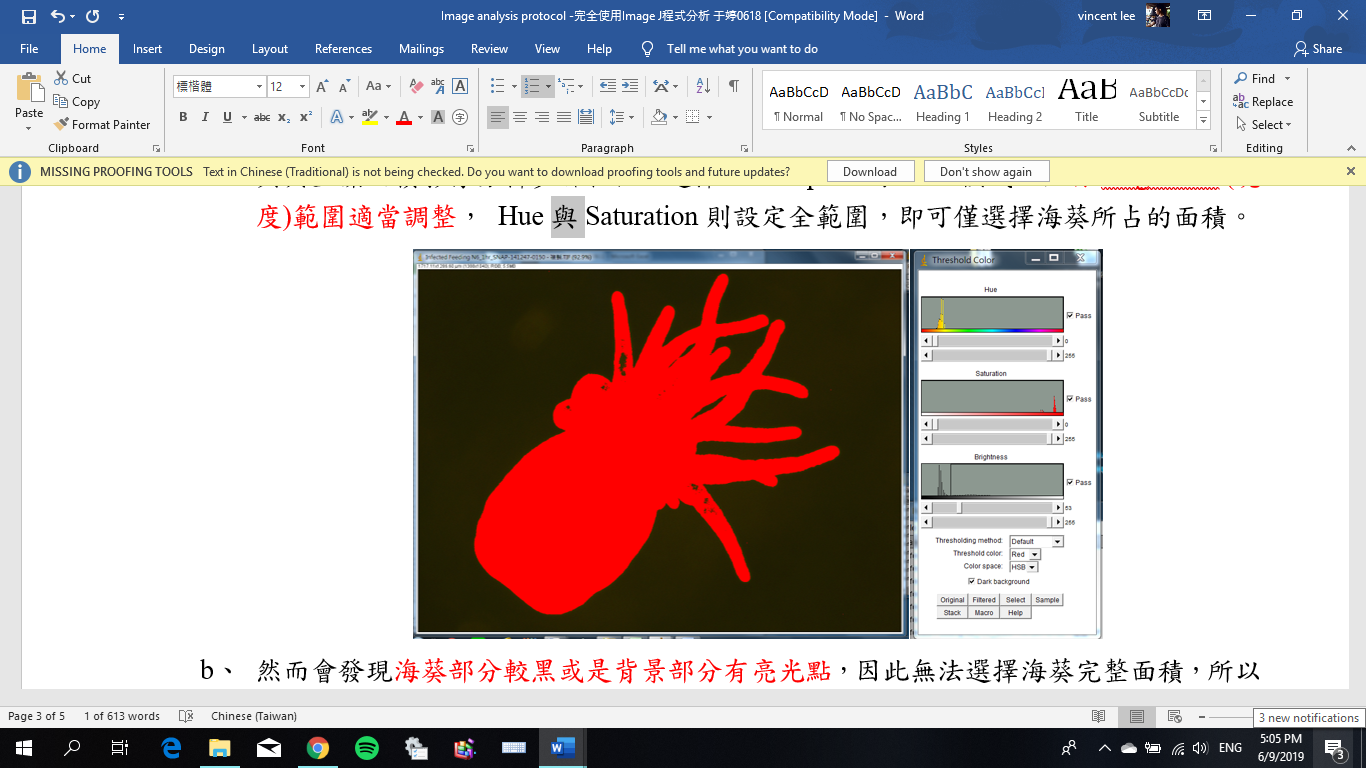


**Figure 8.** Example of a sea anemone whose total area has been highlighted by ImageJ.

1. However, note that several black areas can be seen within the anemones; this suggests incomplete area selection. There are also areas between tentacles that represent empty space but were “called” anemone material by ImageJ.
2. Click “Select” and use the color pinpoint and drawing tools to cover the

unselected areas (i.e., anemone tissues that were not selected) and undesirable areas (non-anemone areas that were inadvertently selected) and save the processed image (Fig. 9).


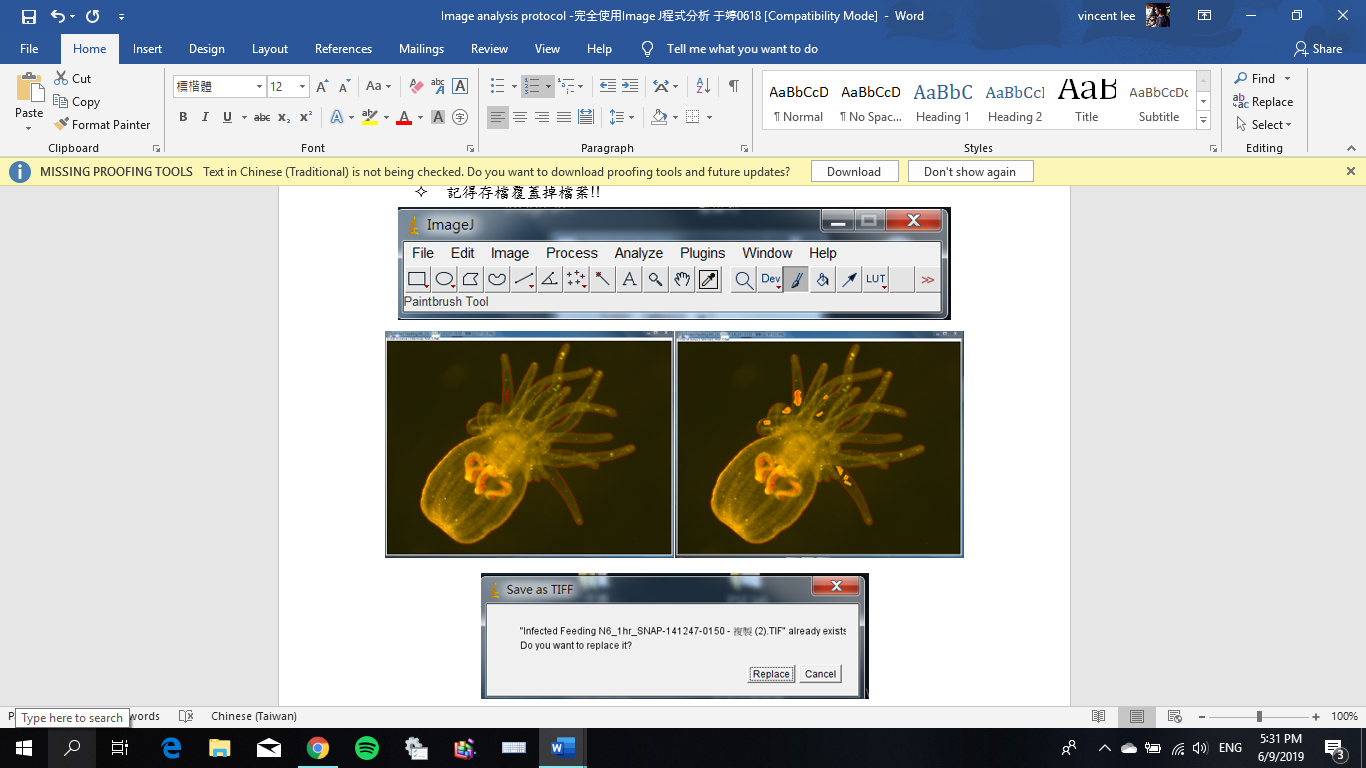


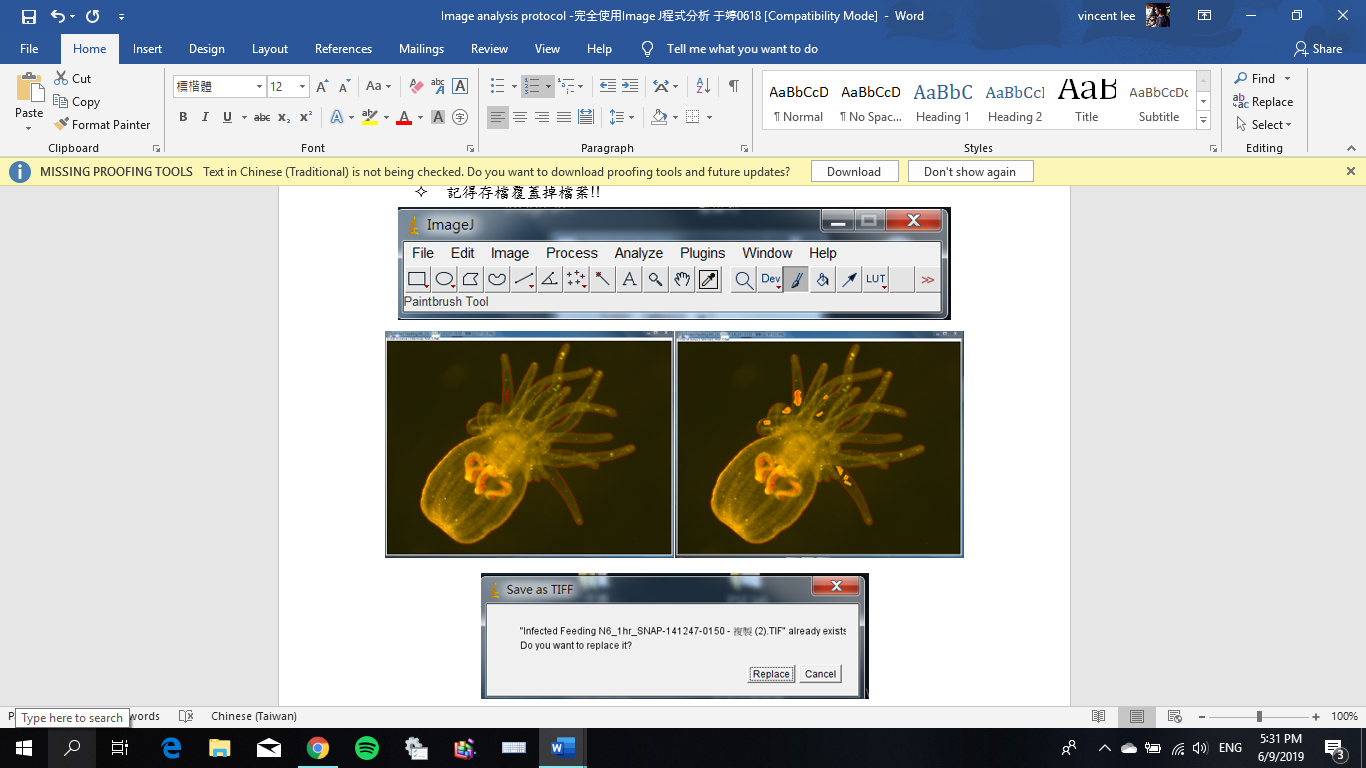


**Figure 9**. Modifying the anemone area borders.

Reopen the image and set the “Brightness” to the previous level. The anemone should now be properly selected in its entirety (Fig. 10).


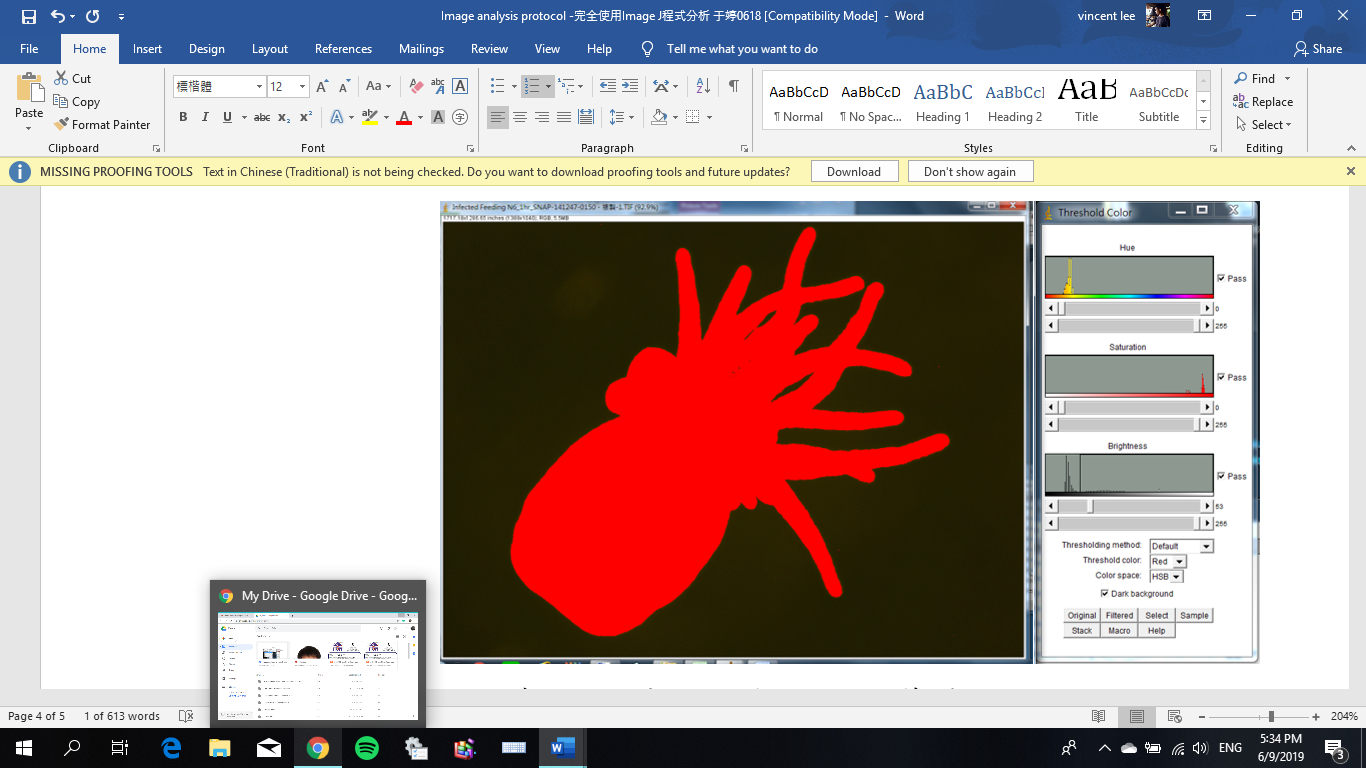

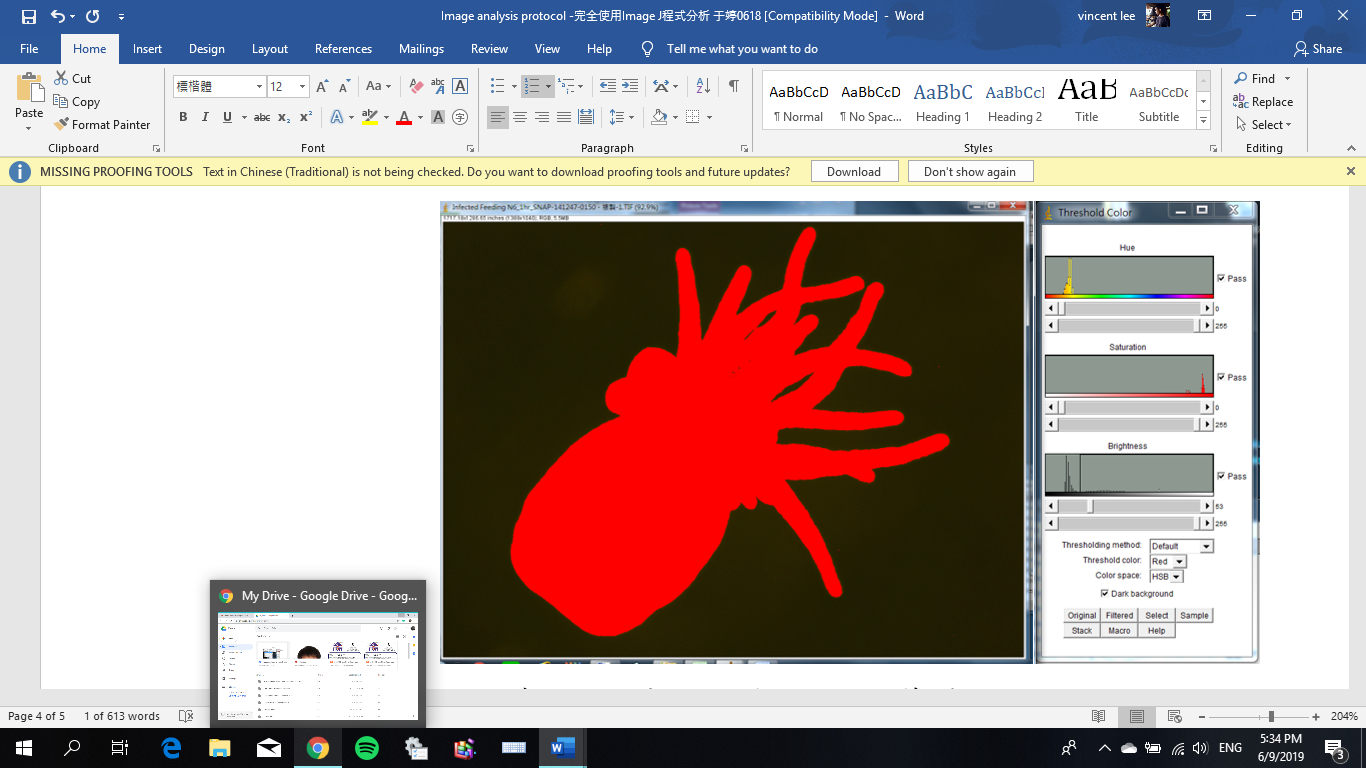


**Figure 10**. Properly outlined and filled anemone image.

1. Click on Analyze → Measure to measure the selected area. Note that the scale bar must be set beforehand).

**【Example】the tissue area of the anemone is 653,457.91 μm^2^**

1. Estimate the number and density of dinoflagellate cells by the following formula:

- The total number of dinoflagellate cells = the total area of dinoflagellate cells in the image **÷** the average area of a single dinoflagellate cell (33.064 μm^2^)*

*The diameter of a single dinoflagellate cell averages 6.49 μm (Pasaribu et al., 2015)

- The density of dinoflagellate cells within an anemone = the total number of dinoflagellate cells ÷ the tissue area of the anemone

**【Example 】 the number of dinoflagellate cells=**

**13,187.83 μm^2^/(33.064 μm^2^/cell)= 398.86 cells**

**【Example 】 the density of dinoflagellate cells within an anemone=**

**398.86 cells/653,457.91 μm^2^ = 0.61 cells/mm^2^**

**References:**

Pasaribu B, Weng LC, Lin IP, Camargo E, Tzen JT, Tsai CH, Ho SL, Lin MR, Wang LH, Chen CS, Jiang PL. 2015. Morphological variability and distinct protein profiles of cultured and endosymbiotic *Symbiodinium* cells isolated from *Exaiptasia pulchella*. Sci Rep. 5:15353. doi: 10.1038/srep15353.

Schneider CA, Rasband WS, Eliceiri KW. 2012. NIH Image to ImageJ: 25 years of image analysis. Nature Methods 9(7): 671-675, PMID 22930834
