# Supplementary figures and images for: A shift away from mutualism under food-deprived conditions in an anemone-dinoflagellate association
Source: PeerJ. 2020 Oct 28;8:e9745. doi: 10.7717/peerj.9745 (PMC7602683; doi:10.7717/peerj.9745)

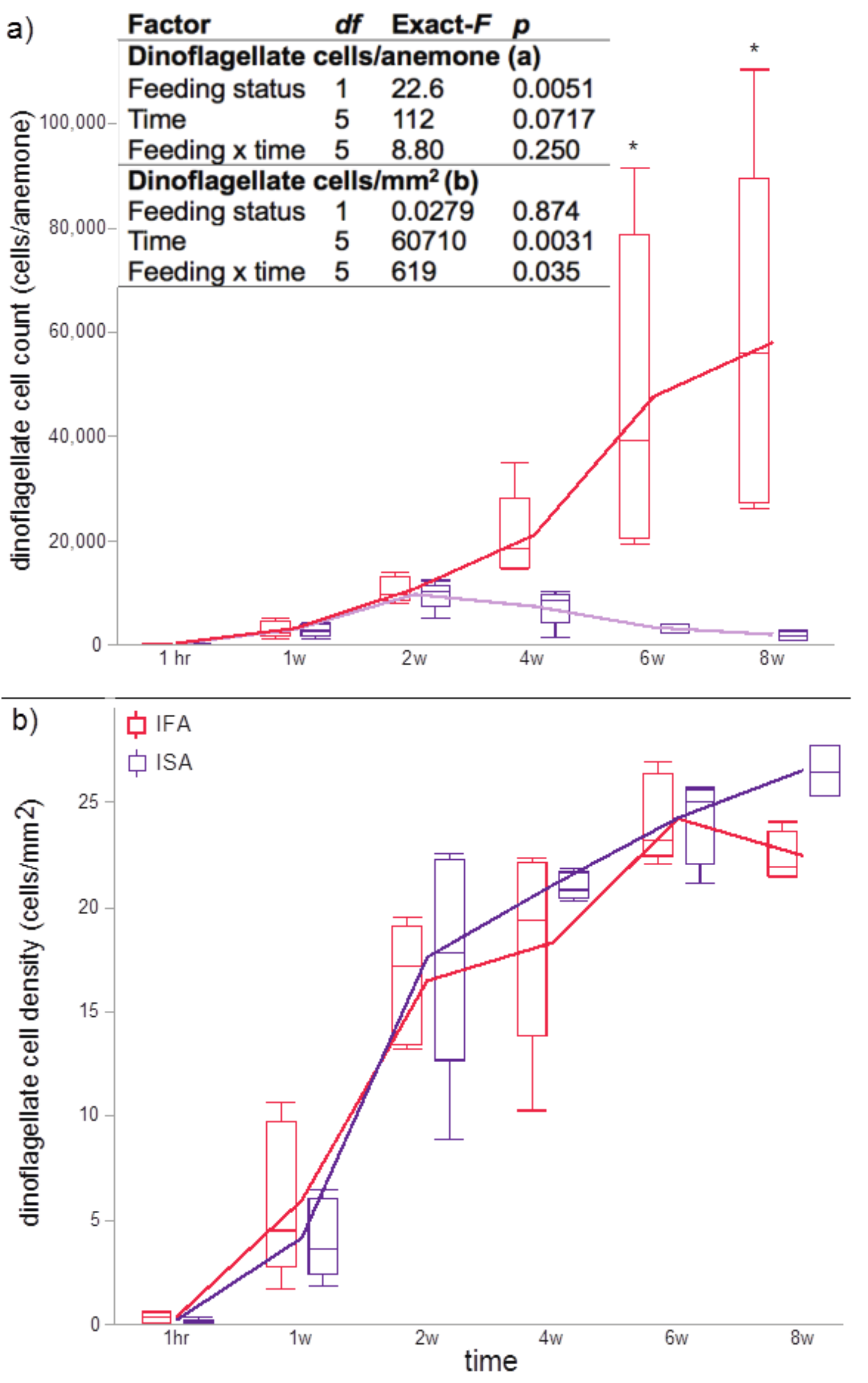

Supplement: Figure S1 — The results of the repeated measures ANOVAs (feeding status over time) have been presented in an inset in (A)(log and untransformed data for cells/anemone and cells/ mm2, respectively). Although there was not a statistically significant interaction effect for the cells/anemone data (A), Tukey’s post-hoc tests nevertheless revealed intra-time treatment differences at the 6- and 8-week (w) sampling times (p < 0.05; denoted by asterisks). [file peerj-08-9745-s003.png]
